# Correction: Intrinsic motivation for choice varies with individual risk attitudes and the controllability of the environment

**DOI:** 10.1371/journal.pcbi.1011599

**Published:** 2023-10-27

**Authors:** Jérôme Munuera, Marta Ribes Agost, David Bendetowicz, Adrien Kérébel, Valérian Chambon, Brian Lau

The fourth author’s name is spelled incorrectly. The correct name is: Adrien Kérébel. The correct citation is: Munuera J, Ribes Agost M, Bendetowicz D, Kérébel A, Chambon V, Lau B (2023) Intrinsic motivation for choice varies with individual risk attitudes and the controllability of the environment. PLoS Comput Biol 19(8): e1010551. https://doi.org/10.1371/journal.pcbi.1010551.
