# Supplementary material for: Intrinsic motivation for choice varies with individual risk attitudes and the controllability of the environment
Source: PLoS Comput Biol. 2023 Aug 11;19(8):e1010551. doi: 10.1371/journal.pcbi.1010551 (PMC10479909; doi:10.1371/journal.pcbi.1010551)
Supplement: S1 Text — (PDF) [file pcbi.1010551.s001.pdf]

Supporting Information for:

Intrinsic motivation for choice varies with individual risk attitudes and the controllability of the environment

Jérôme Munuera, Marta Ribes Agost, David Bendetowicz, Adrien Kerebel, Valérian Chambon, Brian Lau

Correspondence to:
Brian Lau
Email: brian.lau@upmc.fr

This PDF file includes:

S1 Text
S1 Figures A to I
S1 Tables A to B
S1 References

S1 Text

Model and parameter recovery

In order to verify that we could discriminate different TDRL models and accurately estimate parameters, we performed model and parameter recovery analyses on simulated datasets. We considered six key models, the three different value estimates (SARSA, Q-learning, β-pessimistic) with and without a free choice bonus (ρ). For each of these models, we simulated the performance of 5000 virtual subjects using the same set of conditions that real subjects experienced in experiment 1. We explored a wide parameter space, drawing generative parameters from the following distributions:

$$\alpha \sim \text{beta(shape1=1.75, shape2=2.75)}$$
$$1/\tau \ \sim \text{gamma(shape=2.5, scale=1)}$$
$$\beta \sim \text{beta(shape1=1, shape2=2.25)}$$
$$\rho \sim \text{gamma(shape=2.5, scale=0.25)}$$

We fitted simulated data and performed model selection using the same procedures as for real data. From this data, we calculated confusion and inversion matrices(1) (Fig D(A) in S1 Text), which give the probability that the data simulated by one model is best fit by another and the probability that one model is more likely generated by another, respectively. We found that model recovery was good, although Q-learning and SARSA algorithms were confused in the parameter ranges used. This is due to the fact that Q-learning is strictly optimistic by considering only the best future state-action value, whereas SARSA can be more or less optimistic depending on the sensitivity of the mapping from state-action value differences to behavioral policy. Thus, our use of a softmax policy means that SARSA approaches Q-learning as $1/\tau$ increases. Therefore, we treated Q-learning and SARSA algorithms together as "optimistic" in the main text. Pooling these two models together indicates that model recovery is good (Fig D(B) in S1 Text).

We also examined parameter recovery using correlations between estimated and simulated parameters, both with the same parameter as well as across parameters (Fig D(C-D) in S1 Text). We found that parameter recovery was good (diagonal correlations), and parameter confounding was quite weak (off-diagonal correlations).

Free choice bonus compared to biased softmax policy

An alternative model for choice preference would be a bias added to the free state-action Q-value when a 1st-stage decision is made. A key difference between the outcome bonus we implemented versus a biased policy is that the former enters into subsequent value updates to modify Q-values. Importantly, rewards were probabilistically delivered (except for the one deterministic condition in Experiment 1), meaning that a reward-prediction error learning rule could track local stochastic fluctuations in extrinsic rewards that could yield changes in free choice preference. A bias, on the other hand, can generate free choice preference but will not enter directly into subsequent value updates (hence it will not directly affect Q-value learning). So concretely, let a^1 represent the action that selects the free option, then a softmax policy biased towards free choice is:

$$\pi(\mathrm{s}, \mathrm{a}^1) = \frac{\exp\left([\mathrm{Q}(\mathrm{s}, \mathrm{a}^1) + \mathrm{bias}]/\tau\right)}{\exp\left([\mathrm{Q}(\mathrm{s}, \mathrm{a}^1) + \mathrm{bias}]/\tau\right) + \exp\left(\mathrm{Q}(\mathrm{s}, \mathrm{a}^2)/\tau\right)}$$

Implementing a biased softmax policy is potentially useful when the bias on decision making is stable, as is the case for perceptual distractors (e.g., a flanker, see(2)). Our initial decision to implement a free choice outcome bonus was directly motivated by prior experiments showing that cues associated with reward contingencies learned in a free choice context were overvalued compared to cues associated with the same reward contingencies learned in a forced choice context(3,4).

In order to determine whether a bonus or bias better fits our data, we fit 24 additional models (one for each outcome bonus variant, replacing the outcome bonus with a policy bias) to each subject's data from experiments 1 & 2. We performed additional model recovery experiments for bonus and bias models. We found that model confusion, p(fit|sim), was 0.89 for bias when the simulation was bias and 0.75 for bonus when the simulation was bonus. We found that model inversion, p(sim|fit), was 0.78 for bias when the fit was bias and 0.87 for bonus when the fit was bonus). These values are high and in a similar range to those we observed for model recovery of the other parameters of the models we used, indicating that model comparison between bias and bonus models is reliable. First, we directly compared model evidence across all model pairs that were identical except that one was fit with a bonus and the other with a bias (2256 model pairs). This yields a distribution of differences (ΔBIC) with a mean = -7.76. This can be interpreted as an approximate Bayes factor that is strongly in favor of the outcome bonus model (5,6). We next examined the winning model for each subject. For 72% (68/94) of subjects the winning model was the same model that was selected before including the models with bias. Finally, we focused specifically on subjects where the winning model included a bonus or a bias (n = 63). We found that the best models for 59% (37/63) of subjects maintained a free choice outcome bonus when

this parameter was originally included in the winning model, while for 30% (19/63) of subjects the best model switched from including an outcome bonus to including a bias instead. 11% (7/63) of subjects who did not have an outcome bonus originally ended up retaining a bias in the expanded model space. This indicates that the outcome bonus model fits best for most subjects. However, the preceding breakdown was based on ΔBIC of any magnitude and does not capture the strength of evidence. Therefore, we plot the ΔBIC (bonus – bias, lower is better) between the winning model and the next best model with a bonus or a bias (Fig G in S1 Text). This ΔBIC distribution has a mean = -13.8, which is strongly in favor of our original outcome bonus model. Moreover, there were 24/63 subjects where |ΔBIC|>20 (mean = 41.2), which constitutes very strong evidence in favor of the bonus model, versus 5/63 with the same strength of evidence in favor of the biased softmax policy (mean = 17.0). Taken together, these results indicate that a free choice outcome bonus fits our data better than a softmax bias, suggesting that most subjects update free and forced option values rather than applying a static bias.

Q-value initialization

Since we used the softmax decision rule we originally initialized the Q-values at 0 because, like initializing at 0.5, it indicates initial indifference between options (this decision rule considers only Q-value differences). However, initializing at 0.5 might better represent the subjects' priors and therefore provide a better fit to their data. To address this, we fit the whole set of models using initial Q-values = 0.5 (4512 model fits). First, we directly compared model evidence across all models with initial Q-values = 0 and 0.5, which yields a distribution of differences (ΔBIC) with a mean = -17.6. This can be interpreted as an approximate Bayes factor that is strongly in favor of initial Q-values = 0 (5,6). We next examined this more closely by comparing the best model selected for each subject under each initialization (Fig H in S1 Text, left panel). Again, the evidence is in favor of initial Q-values = 0, with a mean difference = -4.38, which can also be observed using Tjur's (7) R^2 to measure model fit, although the average difference in fit is modest (Fig H in S1 Text, right panel). For 63% of subjects (59/94), the model with initial Q-values = 0 is selected (ΔBIC<0). In 40% of the cases (14/35) where the model with initial Q-values = 0.5 is selected (ΔBIC>0), the selected model was structurally identical to that selected for initial Q-values = 0 (i.e., same target policy, presence/absence of free choice bonus, etc.). In the remaining 21/35 subjects, 8 exhibited ΔBIC>6, which constitutes strong evidence (5) in favor of initial Q-values = 0.5. For these, we examined our main parameter of interest, the free choice bonus, and we found that this parameter was retained in every case where it was retained in our initial fits with initial Q-values = 0 (*n* = 6). Finally, we compared the free choice bonus for all models where it was retained for both initial Q-

values = 0 and 0.5 (n = 51) and found that the estimated bonus parameters were significantly correlated (r = 0.838, p < 1e-6). Taken together, these analyses indicate that, under the conditions of our task, the evidence supports our original choice of initial Q-values = 0. However, some subjects were indeed better fit with initial Q-values = 0.5. We therefore selected the best Q-value initialization for each subject.

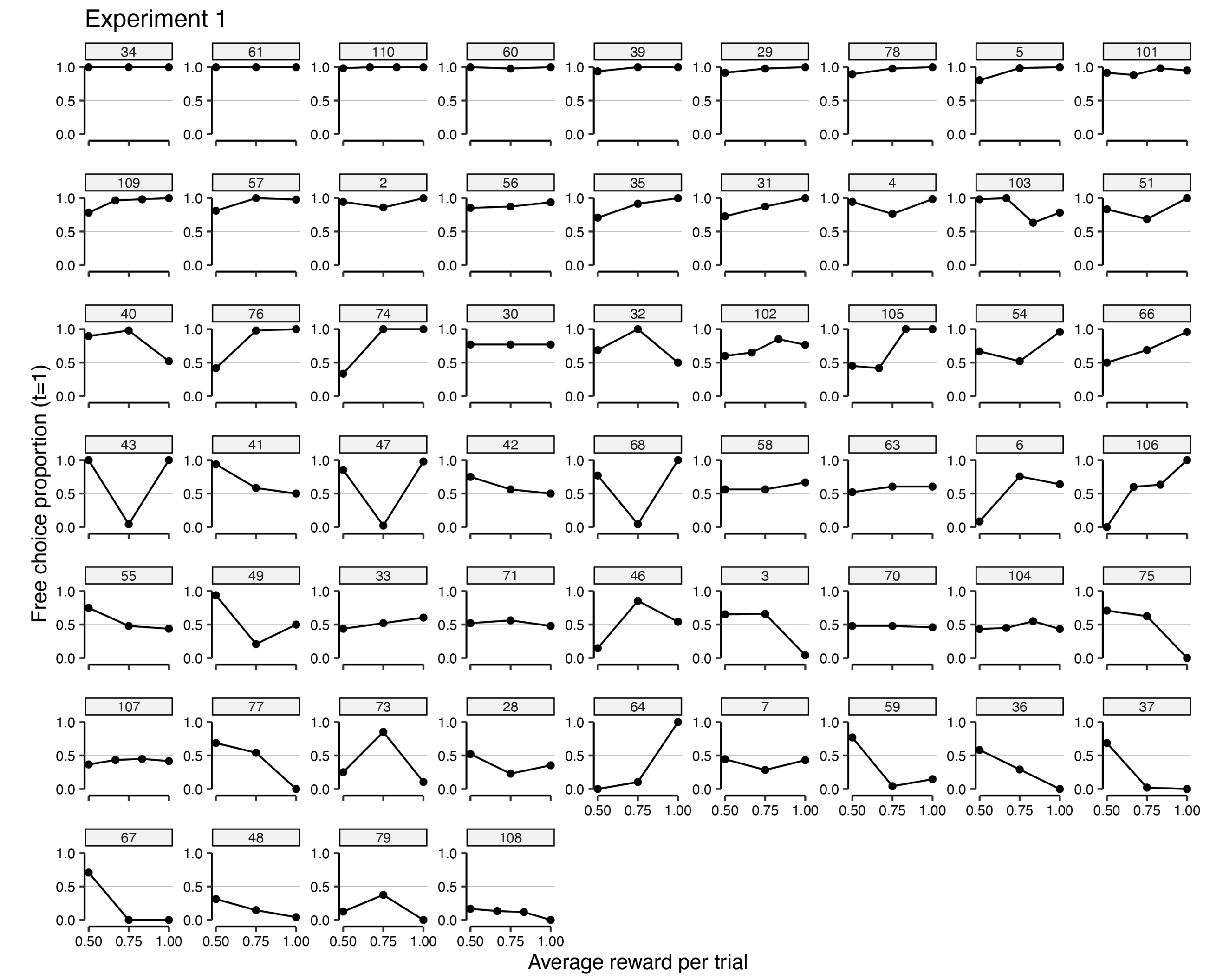

Fig A in S1 Text. First-stage free choice proportions for each subject in experiment 1. Subjects are ordered from left to right, top to bottom according to average free choice proportion (matches ordering of Supplementary Table 1).

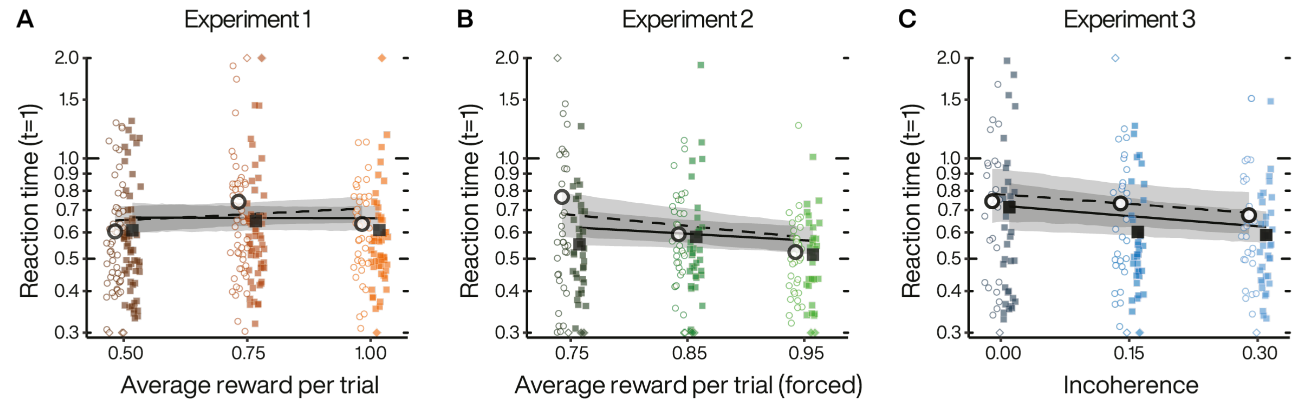

Fig B in S1 Text. First-stage reaction times for the different conditions in each experiment. **A.** Reaction times as a function of extrinsic reward probabilities in experiment 1. Lines indicate the estimated median reaction times with 95% credible intervals for reaction times corresponding to *free* (solid) and *forced* (dashed) selections. Black squares and circles represent the mean of individual subject median reaction times for *free* and *forced* selections respectively. Colored symbols represent individual subject medians. Overall estimated trend = 0.054, 95% CI = [-0.062, 0.175] (p = 0.370). Estimated trend forced = 0.117, 95% CI = [-0.025, 0.259] (p = 0.109). Estimated trend free = -0.009, 95% CI = [-0.141, 0.116] (p = 0.890). **B.** Same as A but as a function of extrinsic reward probabilities of *forced* trials in experiment 2. Overall estimated trend = -0.367, p5% CI = [-0.694, -0.024] (p = 0.020). Estimated trend forced = -0.464, 95% CI = [-0.884, -0.076] (p = 0.016). Estimated trend free = -0.268, 95% CI = [-0.651, 0.091] (p = 0.128). **C.** Same as A and B but as a function of incoherence in experiment 3. Overall estimated trend = -0.279, 95% CI = [-0.570, 0] (p = 0.040). Estimated trend forced = -0.309, 95% CI = [-0.660, 0.0329] (p = 0.060). Estimated trend free = -0.250, 95% CI = [-0.548, 0.024] (p = 0.063).

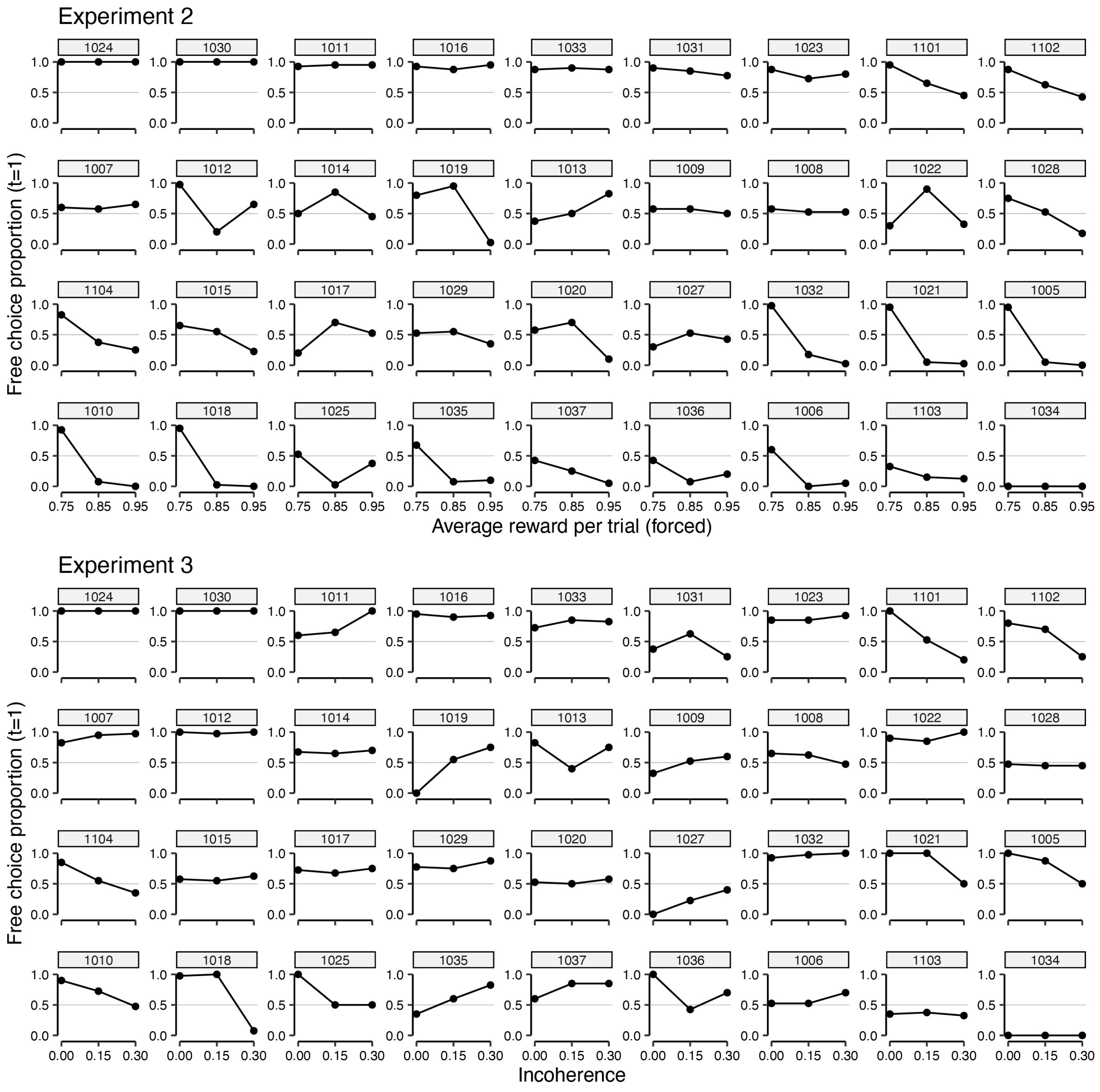

Fig C in S1 Text. First-stage free choice proportions for each subject in experiments 2 (upper panels) and 3 (lower panels). Subjects are ordered from left to right, top to bottom according to average free choice proportion in experiment 2 (matches ordering of Supplementary Table 2).

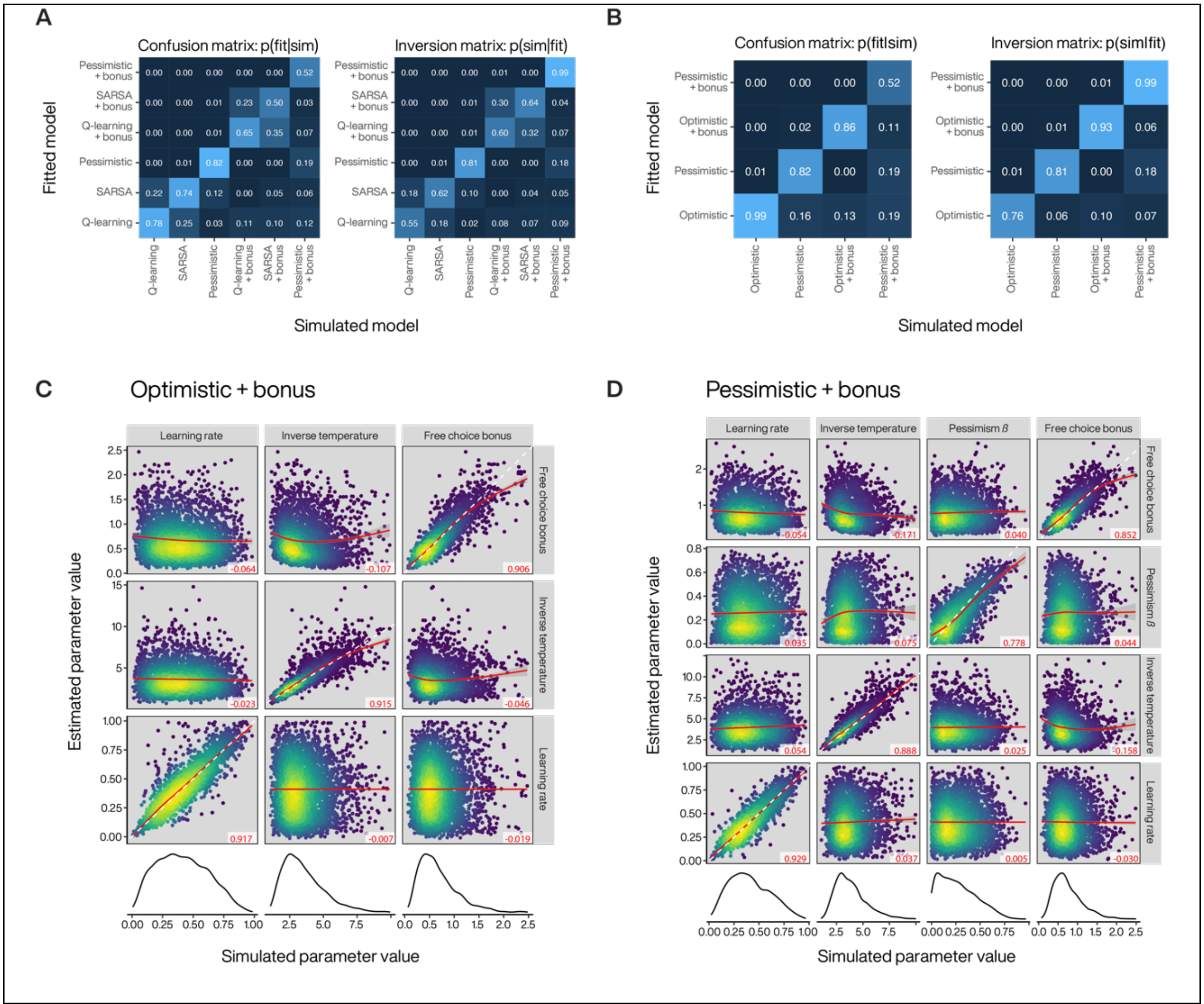

Fig D in S1 Text. Model and parameter recovery simulations. **A.** Confusion and inversion matrices for six models varying value update target (Q-learning, SARSA, or β-pessimistic) and free choice bonus (presence or absence). **B.** Confusion and inversion matrices formed by treating Q-learning and SARSA together as models with "optimistic" value update targets. **C.** Point density plots of simulated and estimated parameter values for models with an optimistic target (both Q-learning and SARSA). The dashed white line in the diagonal panels has unity slope, and the red lines represent an additive model smooth. The red numbers listed in the lower right of each panel are the correlation coefficients for each simulated and estimated parameter. The bottom row contains density estimates for the simulated parameter values. **D.** As C. for β-pessimistic target with a free choice bonus.

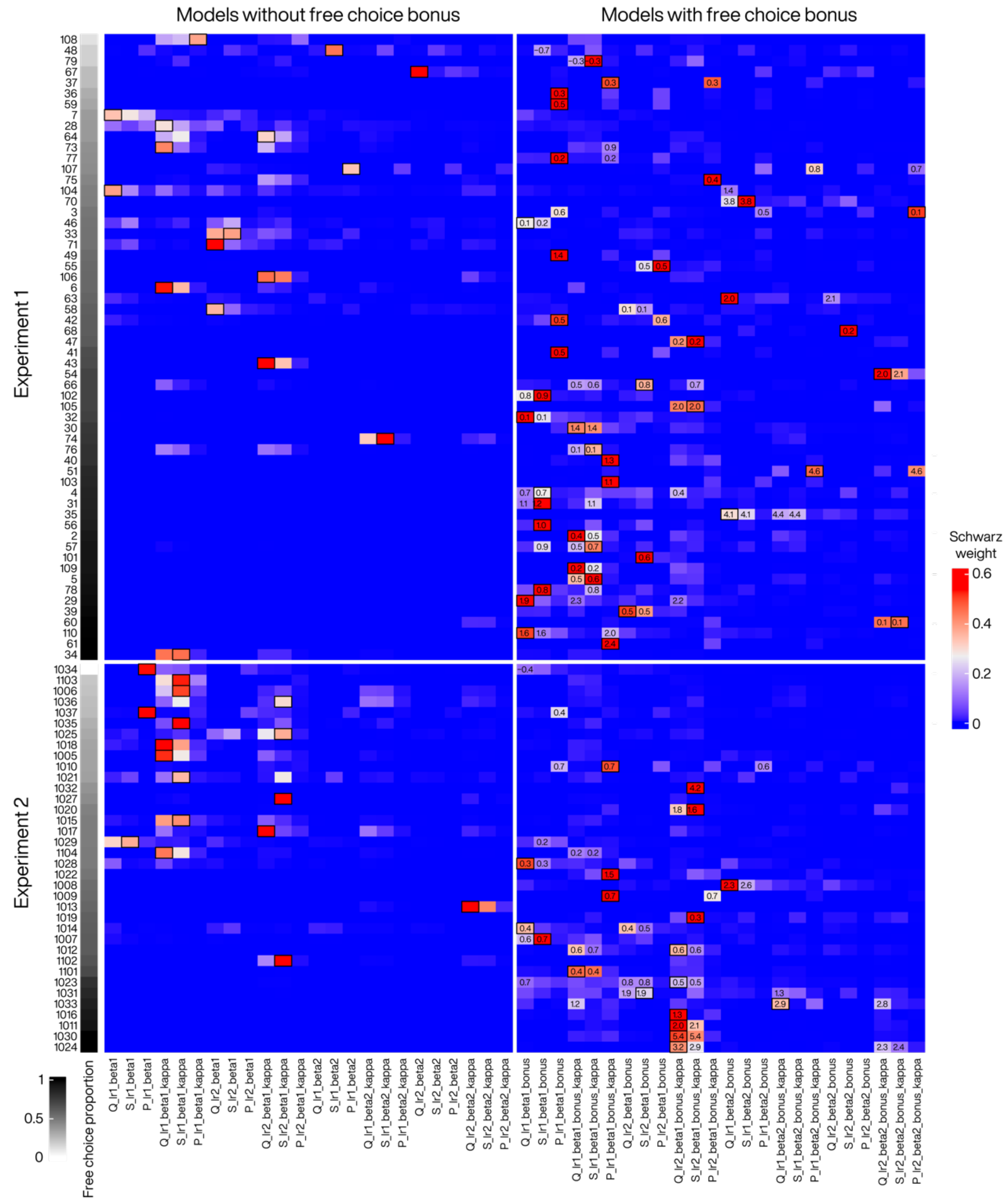

Fig E in S1 Text. Model selection for each subject in experiments 1 (top panels) and 2 (bottom panels). Each row represents the normalized BIC values (Schwarz weights) associated with each of the 48 models fitted to the data of each subject. These sum to one for each subject, and the selected model is highlighted by a black frame. The left panels contain models without a free choice bonus, whereas the right panels contain models with a free choice bonus. The column labels at the bottom give the model specification (target policy is given by the first letter, with Q = off policy maximum, S = SARSA, and P = β-pessimistic policy; lr = learning rate, which if followed by a 2 indicates a separate learning rate for the 1st- and 2nd-stage; beta = softmax temperature, which if followed by a 2 indicates a separate softmax temperature for the 1st- and 2nd-stage; kappa = choice stickiness; bonus = free choice bonus). Numbers in boxes give the free choice bonus (ρ) wherever the Schwarz weight > 0.10). Subjects are arranged from lowest to highest average free choice preference (top to bottom, for each experiment). See Tables A and B in S1 Text for the other parameter estimates of the selected model for each subject.

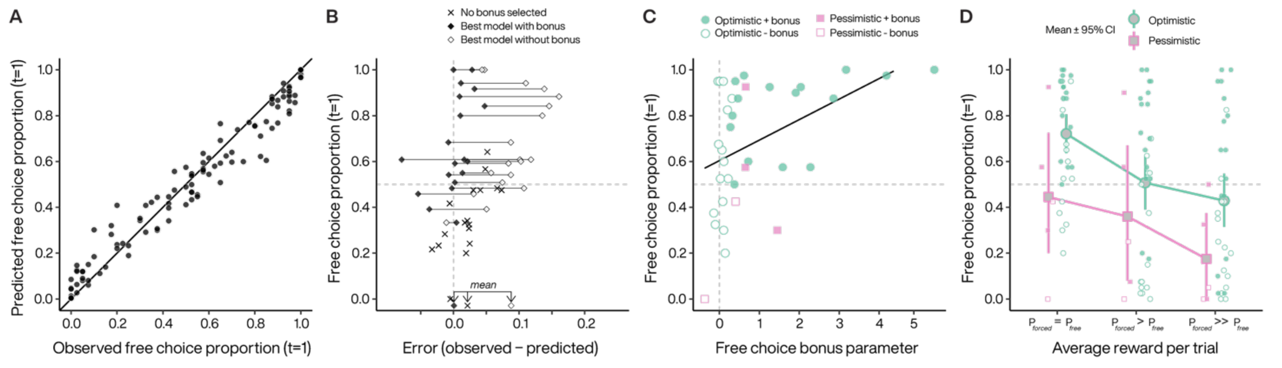

Fig F in S1 Text. Reinforcement learning models capture individual choice behavior in experiment 2. **A.** Free choice proportions predicted by winning model plotted against observed free choice proportions for each condition for each subject. **B.** Obtained free choice proportion as a function of model error, averaged over all conditions. For subjects where the selected model did not include a free choice bonus, only one symbol (X) is plotted. For subjects where the selected model included a free choice bonus, two symbols are plotted. Filled symbol represents the fit error with the selected model, and the open symbol represents the next best model that did not include a free choice bonus. Lines connect individual subjects. **C.** Bonus coefficients increase as a function of subjects' preference for *free* options irrespectively of the target policy they used when performing the task. Choice preference from equal probability blocks (P=0.75). Filled circles indicate that the best model included a free choice bonus parameter. Line illustrates a generalized additive model smooth. **D.** Both optimistic and pessimistic subjects decrease their *free* option preference as the *forced* option value increases. Symbol legend from **C** applies to the small points representing individual means in **D**. Error bars for 95% CI.

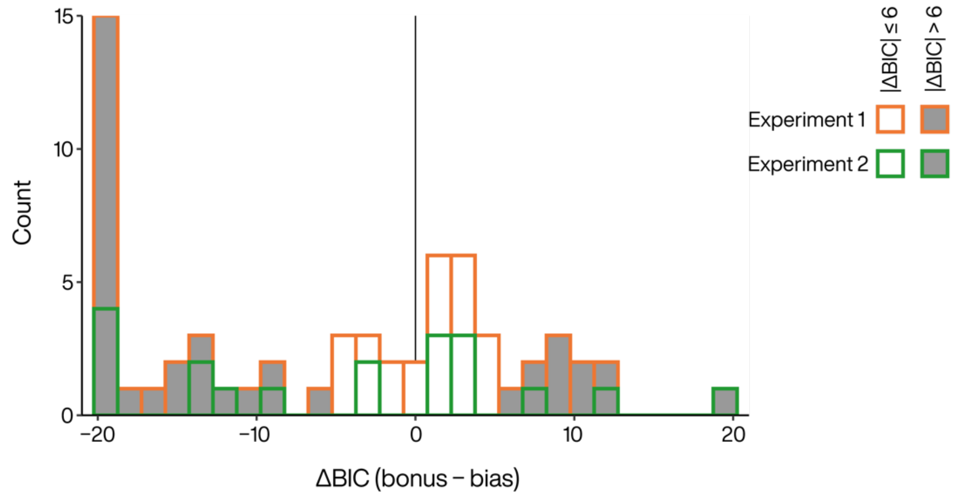

Fig G in S1 Text. Strength of evidence supporting free choice bonus model versus biased softmax policy model. Values along the abscissa represent the difference in BIC scores between the winning model and the next best model with a bonus or a bias for all subjects where the winning model included one of these parameters (*n* = 63). Differences less than -20 are included in the leftmost bin (*n* = 14) and differences greater than 20 are included in the rightmost bin (*n* = 1). Shaded bars indicate data where $|\Delta BIC| > \mathbf{6}$, corresponding to strong evidence in favor of the bonus model (negative values) or the bias model (positive values). See S1 Text (**Free choice bonus compared to biased softmax policy**) for details.

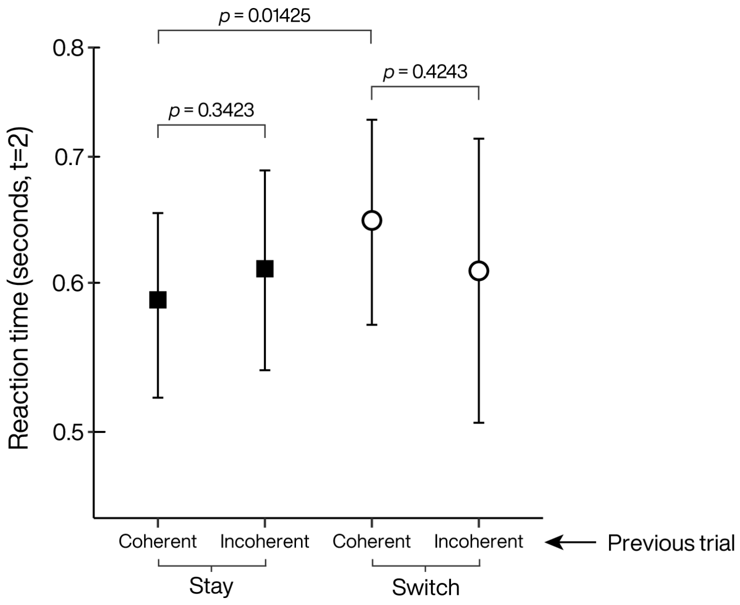

Fig H in S1 Text. Estimated median reaction times in 2nd-stage *free* trials of experiment 3 according to the coherence of the immediately preceding trial and whether subjects stayed (repeated) or switched their fractal selection. We tested whether 2nd-stage reaction times were specifically slowed in repeated selections (stays) of the same fractal target following trials where incoherence was experienced. We observed an estimated median difference (incoherent - coherent) on stays = 22ms (95% CI = [-23, 69]), which was not significant ($p = 0.3423$). Thus, we found no strong evidence for an effect of incoherence on reaction times in repeated selections, suggesting that subjects were not confused by incoherent trials. Consistent with the literature on switch costs, we did find that "switching" was slower than "staying" in coherent trials ($p = 0.014$, median difference [switch – stay] = 59ms, 95% CI = [12, 112]). We fit reaction times using a Bayesian linear mixed model with shifted lognormal errors. Error bars represent 95% credible intervals. We calculated *p*-values by converting the probability of direction, which represents the closest statistical equivalent to a frequentist *p*-value (8).

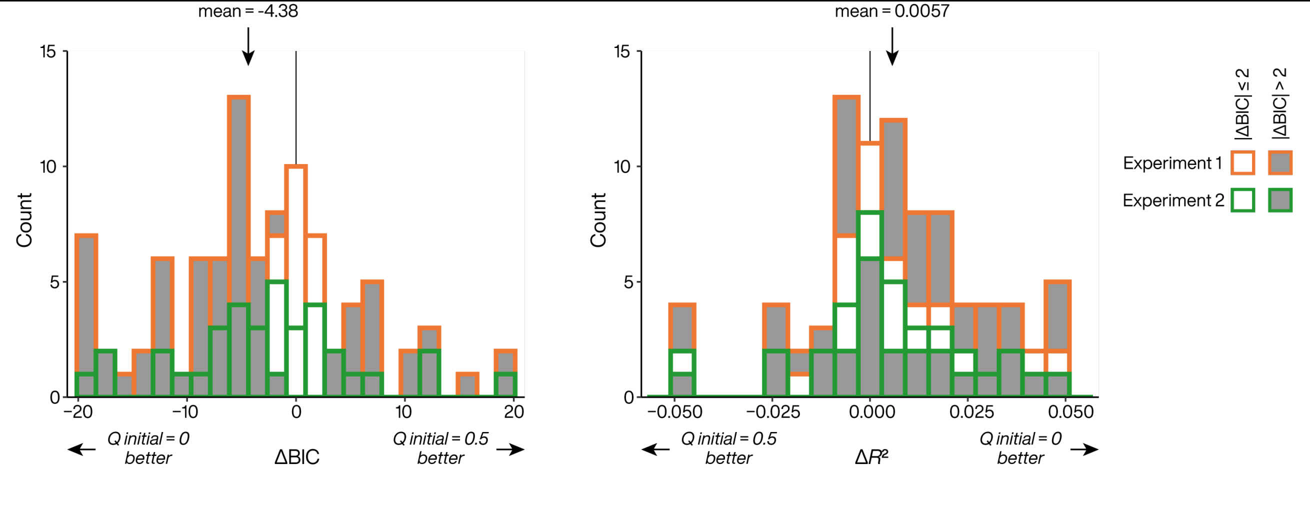

Fig I in S1 Text. Comparing models fit with initial Q-values equal to 0 or 0.5. The left panel shows ΔBIC scores (negative values favor initial Q-values = 0), and right panel shows delta Tjur's R^2(positive values favor initial Q-values = 0).

Experiment 1

Id	Free choice preference	Target policy	Q-value initial-ization	Pessimist weight (β)	Free choice bonus (ρ)	Learning rate (α)	Learning rate, t=1 (α)	Learning rate, t=2 (α)	Softmax ($1/\tau$)	Softmax t=1 ($1/\tau$)	Softmax t=2 ($1/\tau$)	Choice stickiness (κ)
108	0.10	Pessimistic	0	0.75		0.08			7.37			0.20
79	0.17	Optimistic	0		-0.30	0.14			5.31			0.27
48	0.17	Optimistic	0			0.12				12.73	1.97	
37	0.24	Pessimistic	.5	0.03	0.32	0.18			9.91			0.16
67	0.24	Optimistic	0				0.03	0.14		28.99	5.59	
36	0.29	Pessimistic	.5	0.01	0.28	0.23			5.75			
59	0.32	Pessimistic	.5	0.01	0.47	0.19			6.77			
7	0.36	Optimistic	0			0.04			5.69			
28	0.37	Optimistic	0			0.14			9.27			0.06
64	0.37	Optimistic	0				0.23	0.01	12.65			0.14
73	0.40	Optimistic	0			0.07			5.23			0.23
77	0.41	Pessimistic	.5	0.38	0.22	0.09			14.51			
107	0.42	Pessimistic	0	0.02		0.10				0.47	6.81	
75	0.44	Pessimistic	0	0.40	0.43		0.03	0.30	5.68			0.40
104	0.47	Optimistic	0			0.08			4.50			
70	0.47	Optimistic	0		3.84	0.29				0.01	1.95	
3	0.50	Pessimistic	.5	0.75	0.13		0.02	0.05		29.36	10.65	0.00
46	0.51	Optimistic	0		0.12	0.04			6.77			
71	0.52	Optimistic	0				0.00	0.66	2.81			
33	0.52	Optimistic	0				0.01	0.07	5.06			
49	0.55	Pessimistic	0	0.43	1.37	0.59			5.04			
55	0.56	Pessimistic	.5	0.18	0.54		0.01	0.24	7.35			
106	0.56	Optimistic	0				0.01	0.26	8.66			0.17
6	0.56	Optimistic	0			0.03			6.33			0.14
63	0.58	Optimistic	0		2.00	0.48				0.16	1.62	
58	0.60	Optimistic	0				0.03	0.15	5.59			
42	0.60	Pessimistic	0	0.62	0.51	0.34			3.56			
68	0.60	Optimistic	0		0.24		0.04	0.17		13.66	2.28	
47	0.62	Optimistic	0		0.20		0.01	0.09	9.62			0.16
41	0.67	Pessimistic	.5	0.48	0.46	0.11			6.34			
43	0.68	Optimistic	.5				0.82	0.00	46.20			0.05
66	0.72	Optimistic	0		0.84		0.96	0.01	4.01			
54	0.72	Optimistic	0		2.04		0.01	0.59		0.57	2.45	2.22
102	0.72	Optimistic	0		0.95	0.04			2.09			
105	0.72	Optimistic	0		2.02		0.01	0.89	1.93			1.58
32	0.73	Optimistic	.5		0.06	0.06			22.55			
30	0.77	Optimistic	.5		1.42	0.13			2.48			-0.89
74	0.78	Optimistic	.5			0.24				1.14	8.49	1.64
76	0.80	Optimistic	0		0.11	0.07			11.89			0.10
40	0.80	Pessimistic	.5	0.34	1.32	0.11			1.75			0.44
51	0.84	Pessimistic	0	0.90	4.58	0.50				0.42	0.95	0.65
103	0.85	Pessimistic	0	0.60	1.06	0.13			2.54			0.38
4	0.86	Optimistic	0		0.75	0.13			3.02			
31	0.87	Optimistic	0		1.22	0.46			1.94			
35	0.88	Optimistic	0		4.11	0.58				0.52	1.64	
56	0.89	Optimistic	.5		1.00	0.38			2.72			
2	0.92	Optimistic	0		0.45	0.06			4.29			0.38
57	0.93	Optimistic	.5		0.67	0.05			3.98			0.27
109	0.93	Optimistic	.5		0.20	0.02			16.39			0.07
101	0.93	Optimistic	.5		0.61		0.01	0.09	10.53			
5	0.94	Optimistic	0		0.57	0.06			4.28			0.43
78	0.96	Optimistic	.5		0.83	0.47			5.23			
29	0.97	Optimistic	0		1.92	0.08			1.89			
39	0.98	Optimistic	.5		0.53		0.59	0.02	11.71			
60	0.99	Optimistic	.5		0.09		0.01	0.16		5.72	24.77	0.65
110	1.00	Optimistic	.5		1.58	0.40			3.13			
61	1.00	Pessimistic	0	0.54	2.43	0.29			3.64			0.97
34	1.00	Optimistic	.5			0.23			12.44			0.77
**Mean**	0.64			0.40	1.03	0.20	0.16	0.22	7.40	7.81	5.76	0.43
**STD**	0.25			0.29	1.11	0.17	0.30	0.25	7.28	11.09	6.76	0.60

Table A in S1 Text. Parameter estimates for the selected model for each subject in experiment 1. Subjects are arranged from lowest to highest average free choice preference (column 2). Empty cells indicate that the corresponding parameter was not included in the winning model and were ignored for calculating summary statistics.

Experiment 2

Id	Free choice preference	Target policy	Q-value initial-ization	Pessimist weight (β)	Free choice bonus (ρ)	Learning rate (α)	Learning rate, t=1 (α)	Learning rate, t=2 (α)	Softmax ($1/\tau$)	Softmax t=1 ($1/\tau$)	Softmax t=2 ($1/\tau$)	Choice stickiness (κ)
1034	0.00	Pessimistic	0	0.03		0.15			8.44			
1103	0.20	Optimistic	0			0.03			8.14			0.09
1006	0.22	Optimistic	0			0.01			5.12			0.39
1036	0.23	Optimistic	0				0.47	0.11	2.99			0.44
1037	0.24	Pessimistic	.5	0.60		0.47			4.24			
1035	0.28	Optimistic	0			0.32			4.06			0.34
1025	0.31	Optimistic	0				0.03	0.47	6.65			0.10
1018	0.33	Optimistic	.5			0.05			21.57			0.07
1005	0.33	Optimistic	.5			0.05			9.46			0.19
1010	0.33	Pessimistic	.5	0.01	0.66	0.24			6.48			0.19
1021	0.34	Optimistic	0			0.17			6.96			0.20
1032	0.39	Optimistic	0		4.18		0.00	0.76	0.45			4.45
1027	0.42	Optimistic	0				0.00	0.11	4.02			0.49
1020	0.46	Optimistic	0		1.59		0.01	0.94	0.73			1.83
1017	0.48	Optimistic	0				0.25	0.98	2.47			0.84
1015	0.48	Optimistic	0			0.20			3.80			0.22
1029	0.48	Optimistic	.5			0.01			11.15			
1104	0.48	Optimistic	0			0.02			14.75			0.08
1028	0.48	Optimistic	.5		0.28	0.05			4.31			
1022	0.51	Pessimistic	0	0.17	1.45	0.09			1.82			0.73
1008	0.54	Optimistic	0		2.28	0.13				0.12	1.12	
1009	0.55	Pessimistic	.5	0.35	0.65	0.13			5.70			0.19
1013	0.57	Optimistic	.5				0.01	0.19		0.68	4.60	1.12
1019	0.59	Optimistic	.5		0.33		0.38	0.03	7.45			0.11
1014	0.60	Optimistic	.5		0.38	0.63			1.83			
1007	0.61	Optimistic	0		0.72	0.39			0.96			
1012	0.61	Optimistic	0		0.62		0.22	0.72	2.66			0.29
1102	0.64	Optimistic	0				0.00	0.64	3.04			0.51
1101	0.68	Optimistic	.5		0.40	0.05			6.18			0.15
1023	0.80	Optimistic	.5		0.47		0.05	0.58	3.13			0.25
1031	0.84	Optimistic	.5		1.91		0.42	0.00	15.95			
1033	0.88	Optimistic	0		2.86	0.62				0.67	1.48	0.65
1016	0.92	Optimistic	0		1.26		0.57	0.02	2.78			0.35
1011	0.94	Optimistic	0		2.04		0.03	0.48	1.28			0.87
1030	1.00	Optimistic	0		5.38		0.10	0.75	2.40			1.77
1024	1.00	Optimistic	.5		3.17		0.02	0.21	1.35			1.16
**Mean**	0.52			0.23	1.61	0.19	0.16	0.44	5.52	0.49	2.40	0.65
**STD**	0.24			0.25	1.43	0.20	0.20	0.34	4.74	0.32	1.92	0.88

Table B in S1 Text. Parameter estimates for the selected model for each subject in experiment 2. Subjects are arranged from lowest to highest average free choice preference (column 2). Empty cells indicate that the corresponding parameter was not included in the winning model and were ignored for calculating summary statistics.

SI References

1. Wilson RC, Collins AGE. Ten simple rules for the computational modeling of behavioral data. Elife. 2019 Nov 1;8.
2. Sidarus N, Palminteri S, Chambon V. Cost-benefit trade-offs in decision-making and learning. PLoS Comput Biol [Internet]. 2019 [cited 2022 Jun 13];15(9). Available from: https://pubmed.ncbi.nlm.nih.gov/31490934/
3. Cockburn J, Collins AGE, Frank MJ. A Reinforcement Learning Mechanism Responsible for the Valuation of Free Choice. Neuron. 2014;
4. Chambon V, Théro H, Vidal M, Vandendriessche H, Haggard P, Palminteri S. Information about action outcomes differentially affects learning from self-determined versus imposed choices. Nat Hum Behav. 2020;
5. Kass RE, Raftery AE. Bayes Factors. J Am Stat Assoc. 1995;90(430):773–95.
6. Cavanaugh JE. Model Selection: Bayesian Information Criterion. Wiley StatsRef Stat Ref Online [Internet]. 2016 Feb 15 [cited 2023 Feb 21];1–3. Available from: https://onlinelibrary.wiley.com/doi/full/10.1002/9781118445112.stat00247.pub2
7. Tjur T. Coefficients of determination in logistic regression models—A new proposal: The coefficient of discrimination. Am Stat. 2009;63(4):366–72.
8. Makowski D, Ben-Shachar MS, Chen SHA, Lüdecke D. Indices of Effect Existence and Significance in the Bayesian Framework. Front Psychol. 2019 Dec 10;10:2767.
