# Supplementary material for: Intrinsic motivation for choice varies with individual risk attitudes and the controllability of the environment
Source: PLoS Comput Biol. 2023 Aug 11;19(8):e1010551. doi: 10.1371/journal.pcbi.1010551 (PMC10479909; doi:10.1371/journal.pcbi.1010551)
Supplement: Attachment — Submitted filename: Munuera_etal_ResponsesToReviewers.pdf [file pcbi.1010551.s002.pdf]

We would like to thank all the reviewers for their insightful and constructive questions and comments. We have endeavored to address all their remarks in the revised manuscript and the detailed responses below. We believe that we have addressed all reviewer criticisms and feel that the new data and analyses have substantially clarified, enriched and improved our revision.

All our responses are in italics.

<u>Reviewer #1:</u> Title: Choice seeking is motivated by the intrinsic need for personal control

In this paper, the authors carefully investigate whether choice-seeking is intrinsically motivating. Across three experiments, they show that people have a preference for free choice, even when free choice does not have greater relative value than forced choice. The authors use computational modeling to explore this behavior further, and the winning variation of their model could explain individual differences in choice seeking. The paper explores a classic topic using a modern framework, and is likely of interest to a broad audience. The thoughtful experimental designs are a particular strength of this paper. I have some comments and questions below that I think it would be useful for the authors to clarify/address.

1. One of the primary strengths of this paper is the experimental design/behavioral analyses. Therefore, it will be especially important for the authors to make certain that the complicated task designs are clearly explained.
a. For example, while it is discussed in the main text, there is no explanation of what the red "V" is in the Figure 1 caption. In addition, it wasn't clear to me the purpose of both the red "V" and the black arrows, which both seem to indicate participant (or computer) selection.

We thank the reviewer for pointing out this lack of clarity. The red "V" was associated with the target selected by the subject on each trial. It served as feedback to the subject about their target selection. The red "V" was therefore visible each time the subjects pressed the left or right keys to select a target in the 1st and 2nd-stages. We only used the black arrow in Figure 1 to illustrate a hypothetical subject selection (where the red V is displayed on the screen as the reviewer noticed). We realize that the black arrow is potentially confusing for the reader, and we have removed it from Figure 1. We have also added text in the legend of this figure describing the purpose of the red 'V' (pp.5-6) and clarified the explanation in the Materials and methods (p. 34).

b. In Experiment 3, I assume the black arrows/red "V" on incoherent trials simply point to the participant's perceived selection, but I don't think this is ever mentioned in the text.

Thanks for pointing this out. In experiment 3 the V-shaped was initially light red above the fractal target selected by the subjects then turned dark red (i.e., same color as in experiments 1 and 2) above the final target ultimately selected by the computer. Thus, in coherent trials the light and dark red "V"s overlapped while in incoherent trials they did not. We noted this in the original methods section (p.27, LL.527-531), but to make this difference clearer we have added text to the legend of (new) Figure 6A (p.18) and have added details in the methods section when introducing experiment 3 (p.32-33 and 34).

c. Were participants paid additional compensation depending on their task earnings/performance? This should be mentioned in the text regardless of whether they were or not.

We paid all subjects 40 euros for participating (LL. 449-450 of original manuscript). Subjects were informed that their base compensation would be 30 euros and that an additional compensation of 10 euros would be added if they performed the task correctly. However, correct task performance consisted of successfully completing all trials, and all subjects were paid the additional 10 euros. Thus, compensation was not directly proportional to task earnings, although we instructed subjects that the goal of the task was to gain the maximum number of rewards (large green euro) (LL.461-462 of original manuscript). As part of the exit questionnaire, we asked subjects whether they were

motivated by this additional compensation. Only 12% reported not being motivated by it. We now point this out in the revised manuscript (p.30).

d. On Line 602, the authors state, "Data from the training phase consisted of 2nd-stage actions and rewards, but we also presented subjects with the 1st-stage cues corresponding to the condition being trained (forced or free)." This is the first mention that 1st stage cues are shown during the 2nd stage choice phase. It would be helpful if this was clarified earlier in the manuscript (either in Figure 1, the Methods section, or both).

Thanks for pointing out this potential source of confusion. Each trial was clearly divided into two separate stages, and the 1st-stage targets were never presented during the 2nd-stage of each trial. What we meant in line 602 is that before selecting fractal targets in the 2nd-stage, subjects viewed free or forced targets of the 1st stage since the task was designed in a way that they must pass through the first stage before entering the 2nd-stage. During the training phase, only one of the free or forced targets was displayed on the screen and the subjects could not choose between them (Fig. 1B). We now make this point clearer and earlier in the legend of Figure 1 (pp. 5-6), in the methods section ("Trial structure", p.34), and have adjusted the sentence the reviewer highlights (p.37 of revision).

2. Figure 2D: It's not clear to me why in the "High" panel, there's only data for 24 trials. If this is simply a case of me missing the explanation in the text, then perhaps it would be helpful to reference the figure in the text where this is explained.

The explanation was briefly mentioned in the methods section (LL.511-512 of the original manuscript). In pilot experiments, we observed that subjects learned to select the rewarded target extremely rapidly in the deterministic block (P=1), reaching plateau after ~10 trials. To minimize the duration of the experimental sessions, we chose to reduce the number of training trials to 24 for the deterministic block (instead of 48 as for the other reward contingencies). We note that even with only 24 training trials, acquisition of the reward contingency in the deterministic block was excellent, with most subjects choosing exclusively the rewarded target during the test phase (see Fig. 2E, right panel). We now make this information explicit in the revised manuscript (p.33) and refer to it in the legend of figure 2 (p.7).

3. I appreciated the clever controllability manipulation in Experiment 3. The authors explain that the repetition of free choices across incoherent trials suggests that participants are trying to "regain control." However, I would imagine that this manipulation could cause the participants some confusion. In other words, pressing a "left" button and getting a "right" response probably seems like a bug or an error, and they might just be repeating their actions to test whether they have actually lost control. Because there typically isn't any uncertainty around an action like a key press and a response, I'm wondering whether participants reported being confused by this manipulation, and also whether there were RT differences for repeated choices after incoherent trials.

We thank the reviewer for pointing out this possibility. Confusion was a problem we were also worried about when planning this experiment. Although we did not ask subjects explicitly about confusion, we did implement several experimental precautions to minimize potential confusion. First, incoherent trials could occur following selection of both free and forced options. This was to ensure that subjects did not perceive incoherence as a feature specific to the free choice option (mentioned in Fig. 5 pp.16-17 and p.26, LL.498-504 ("Experimental conditions") of original manuscript) Second, we explicitly informed subjects before experiment 3 that the computers had been extensively tested for any malfunction (both verbally and in instructions displayed on screen). This was to ensure that incoherent trials would not be attributed to a software bug. We now point this out on pp.31-33 ("Experimental conditions") of the revised manuscript. Third, to ensure that target swaps would not be perceived as their own errors (i.e., pressing right when they planned to press left), we manipulated the timing and color of the red V-shape to inform subjects about their 2nd-stage selection. This feedback was initially light red (for 250ms) above the actual fractal target selected by the subjects. Then a dark red V-shape appeared for 750ms at a potentially different location (the same color as

experiments 1 and 2). So, on incoherent trials the light red V-shape highlighting the selected target disappeared after 250ms, and simultaneously a dark red V-shape appeared over the other target for 750ms. This provided visual feedback to inform subjects that they did indeed correctly select one of the keys, but that it was the computer that ultimately produced a target swap. This information was presented on p.27 of the original manuscript, but it was very brief. We have adjusted the text to reemphasize more clearly this point, pp.32-33 and p.34 of the new version. Finally, a unique feature of our experiments was the training phase, where subjects learned 2nd-stage contingencies. In experiment 3, the training phase allowed subjects opportunities to experience and become familiar with incoherent trials.

We thank the reviewer for suggesting the reaction time analysis, which we have performed. One might expect that 2nd-stage reaction times are specifically slowed in repeated selections (stays) of the same fractal target following *trials where incoherence was experienced (Figure R1). We observed an estimated median difference (incoherent - coherent) on stays = 22ms (95% CI = [-23, 69]), which was not significantly different (p = 0.3423). Thus, we do not find strong evidence for an effect of incoherence on reaction times in repeated selections, which suggests that subjects were not confused by incoherent trials. Consistent with the literature on switch costs, we did find that switching was slower than staying on coherent trials (p = 0.014, median difference [switch – stay] = 59ms, 95% CI = [12, 112]).*

Taking the reaction time results together with our explicit instructions, feedback, and training protocol, we believe that subjects were not confused following incoherent trials. We would be happy to integrate Figure R1 and associated results into the supplementary materials if the reviewer thinks it appropriate.

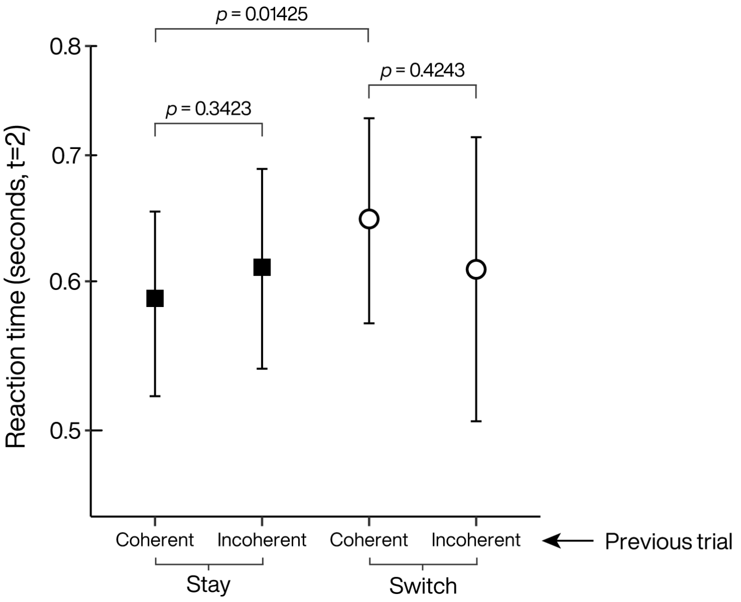

Figure R1. Estimated median reaction times in 2nd-stage *free* trials of experiment 3 according to the coherence of the immediately preceding trial and whether subjects stayed (repeated) or switched their fractal selection. We fit reaction times using a Bayesian linear mixed model with shifted lognormal errors. Error bars represent 95% credible intervals. We calculated *p*-values by converting the probability of direction, which represents the closest statistical equivalent to a frequentist *p*-value (Makowski et al., Front in Psych, 2019).

4. In the context of Experiment 3, the authors talk about manipulating "perceived controllability." I think it is worth being cautious of the language here. "Perceived" controllability implies that participants both explicitly reported a lack of control and yet truly had control over the choice. Instead, it might be best to simply talk about this as a manipulation of the actual controllability (which is what I believe it is, if I understand the task correctly).

The reviewer is right, we did manipulate actual controllability, and we did not ask subjects to explicitly report their perception of controllability. We originally used "perceived" controllability to refer to the fact that controllability in our experiment is illusory since the extrinsic reward probabilities were equal between all terminal states and did not vary with incoherence. However, we agree that this is not precise, and have now removed "perceived" and refer to only "controllability".

5. The authors mention that they pooled two subject groups for Experiments 1a and 1b and that they did not have any "substantial differences." It is important to include the actual evidence that these two subject groups did not differ behaviorally.

We thank the reviewer for pointing out this imprecision. We have included the following text to explain why we pooled data (p.32 of revised manuscript):

"We used a GLMM to compare subjects who experienced four blocks (mean choice preference = 66%) to those that experienced three blocks (mean choice preference = 64%). There was no main effect for group, nor a significant interaction with reward probability (χ^2 = 0.093, $p > 0.05$), and choice preference was significantly greater than 50% for both groups (χ^2 = 1.28, $p > 0.05$). Therefore, we pooled data from these groups for analyses."

6. Beginning on Line 312, the authors write: "Consistent with previous studies, subjects preferred the opportunity to make a choice when expected rewards were equal between terminal actions (P = 0.5)." It would be helpful to cite some of this previous work here.

We have now inserted appropriate references for this sentence (Suzuki 1997; Leotti 2011; i.e., references 2 and 4). This appears on p.22 of the revised manuscript.

7. The authors should consider including a table in the supplement with each model name, brief details of the model (i.e., its parameters), and model fit values.

We have now added significantly more information about the models in the revised manuscript (new Supplementary Fig 5 and Supplementary Tables 1 and 2). These contain the information requested, including specific model names, parameter estimates, and summaries of parameter estimates (bottom two rows of each table). We include these below for convenience.

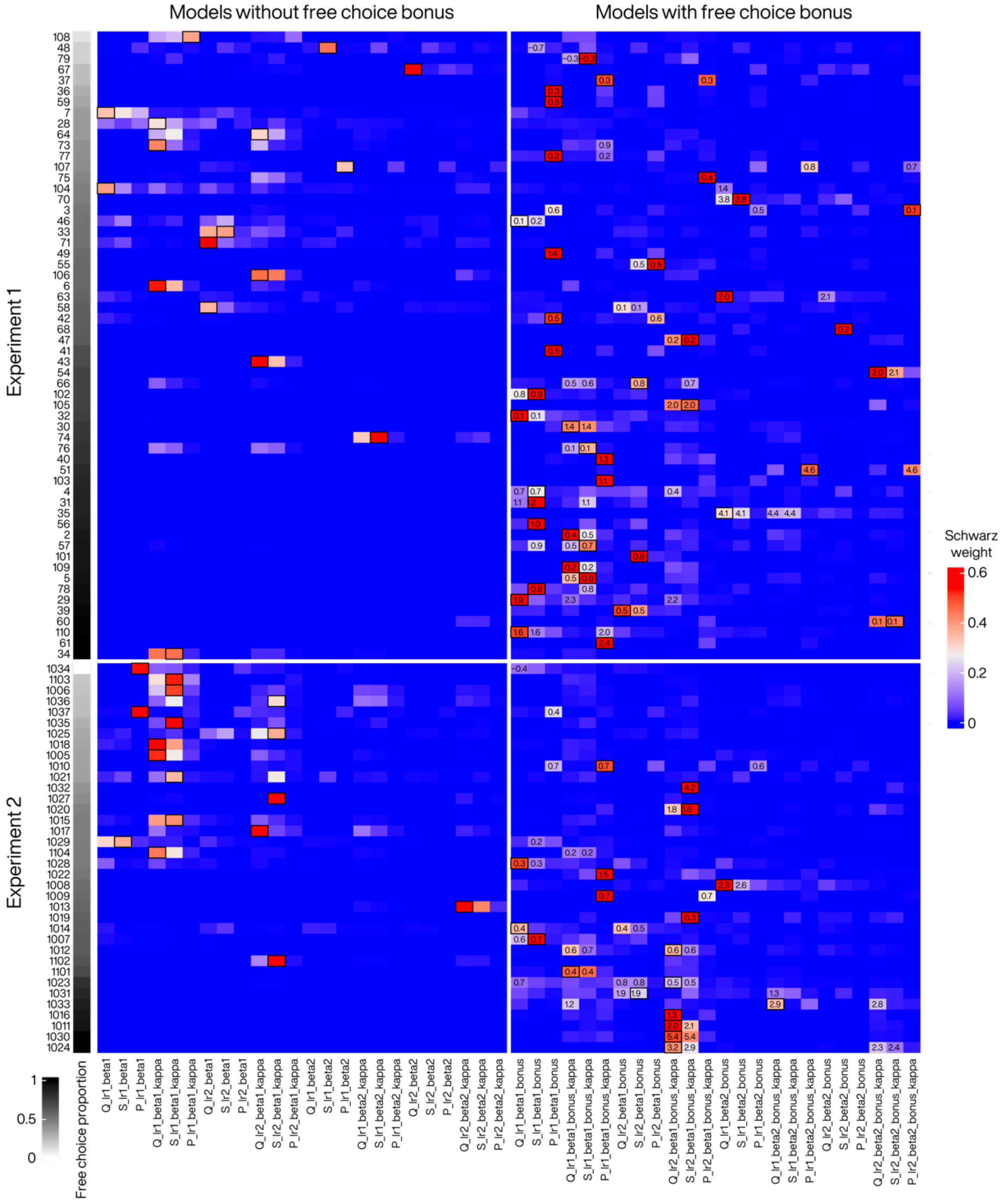

Supplementary Figure 5. Model selection for each subject in experiments 1 (top panels) and 2 (bottom panels). Each row represents the normalized BIC values (Schwarz weights) associated with each of the 48 models fitted to the data of each subject. These sum to one for each subject, and the selected model is highlighted by a black frame. The left panels contain models without a free choice bonus, whereas the right panels contain models with a free choice bonus. The column labels at the bottom give the model specification (target policy is given by the first letter, with Q = off policy maximum, S = SARSA, and P = β-pessimistic policy; lr = learning rate, which if followed by a 2 indicates a separate learning rate for the 1st- and 2nd-stage; beta = softmax temperature, which if followed by a 2 indicates a separate softmax temperature for the 1st- and 2nd-stage; kappa = choice stickiness; bonus = free choice bonus). Numbers in boxes give the free choice bonus (ρ) wherever the Schwarz weight > 0.10). Subjects are arranged from lowest to highest average free choice preference (top to bottom, for each experiment). See Supplementary Tables 1 and 2 for the other parameter estimates of the selected model for each subject.

Experiment 1

Id	Free choice preference	Target policy	Q-value initial-ization	Pessimist weight (β)	Free choice bonus (ρ)	Learning rate (α)	Learning rate, t=1 (α)	Learning rate, t=2 (α)	Softmax ($1/\tau$)	Softmax t=1 ($1/\tau$)	Softmax t=2 ($1/\tau$)	Choice stickiness (κ)
108	0.10	Pessimistic	0	0.75		0.08			7.37			0.20
79	0.17	Optimistic	0		-0.30	0.14			5.31			0.27
48	0.17	Optimistic	0			0.12				12.73	1.97	
37	0.24	Pessimistic	.5	0.03	0.32	0.18			9.91			0.16
67	0.24	Optimistic	0				0.03	0.14		28.99	5.59	
36	0.29	Pessimistic	.5	0.01	0.28	0.23			5.75			
59	0.32	Pessimistic	.5	0.01	0.47	0.19			6.77			
7	0.36	Optimistic	0			0.04			5.69			
28	0.37	Optimistic	0			0.14			9.27			0.06
64	0.37	Optimistic	0				0.23	0.01	12.65			0.14
73	0.40	Optimistic	0			0.07			5.23			0.23
77	0.41	Pessimistic	.5	0.38	0.22	0.09			14.51			
107	0.42	Pessimistic	0	0.02		0.10				0.47	6.81	
75	0.44	Pessimistic	0	0.40	0.43		0.03	0.30	5.68			0.40
104	0.47	Optimistic	0			0.08			4.50			
70	0.47	Optimistic	0		3.84	0.29				0.01	1.95	
3	0.50	Pessimistic	.5	0.75	0.13		0.02	0.05		29.36	10.65	0.00
46	0.51	Optimistic	0		0.12	0.04			6.77			
71	0.52	Optimistic	0				0.00	0.66	2.81			
33	0.52	Optimistic	0				0.01	0.07	5.06			
49	0.55	Pessimistic	0	0.43	1.37	0.59			5.04			
55	0.56	Pessimistic	.5	0.18	0.54		0.01	0.24	7.35			
106	0.56	Optimistic	0				0.01	0.26	8.66			0.17
6	0.56	Optimistic	0			0.03			6.33			0.14
63	0.58	Optimistic	0		2.00	0.48				0.16	1.62	
58	0.60	Optimistic	0				0.03	0.15	5.59			
42	0.60	Pessimistic	0	0.62	0.51	0.34			3.56			
68	0.60	Optimistic	0		0.24		0.04	0.17		13.66	2.28	
47	0.62	Optimistic	0		0.20		0.01	0.09	9.62			0.16
41	0.67	Pessimistic	.5	0.48	0.46	0.11			6.34			
43	0.68	Optimistic	.5				0.82	0.00	46.20			0.05
66	0.72	Optimistic	0		0.84		0.96	0.01	4.01			
54	0.72	Optimistic	0		2.04		0.01	0.59		0.57	2.45	2.22
102	0.72	Optimistic	0		0.95	0.04			2.09			
105	0.72	Optimistic	0		2.02		0.01	0.89	1.93			1.58
32	0.73	Optimistic	.5		0.06	0.06			22.55			
30	0.77	Optimistic	.5		1.42	0.13			2.48			-0.89
74	0.78	Optimistic	.5			0.24				1.14	8.49	1.64
76	0.80	Optimistic	0		0.11	0.07			11.89			0.10
40	0.80	Pessimistic	.5	0.34	1.32	0.11			1.75			0.44
51	0.84	Pessimistic	0	0.90	4.58	0.50				0.42	0.95	0.65
103	0.85	Pessimistic	0	0.60	1.06	0.13			2.54			0.38
4	0.86	Optimistic	0		0.75	0.13			3.02			
31	0.87	Optimistic	0		1.22	0.46			1.94			
35	0.88	Optimistic	0		4.11	0.58				0.52	1.64	
56	0.89	Optimistic	.5		1.00	0.38			2.72			
2	0.92	Optimistic	0		0.45	0.06			4.29			0.38
57	0.93	Optimistic	.5		0.67	0.05			3.98			0.27
109	0.93	Optimistic	.5		0.20	0.02			16.39			0.07
101	0.93	Optimistic	.5		0.61		0.01	0.09	10.53			
5	0.94	Optimistic	0		0.57	0.06			4.28			0.43
78	0.96	Optimistic	.5		0.83	0.47			5.23			
29	0.97	Optimistic	0		1.92	0.08			1.89			
39	0.98	Optimistic	.5		0.53		0.59	0.02	11.71			
60	0.99	Optimistic	.5		0.09		0.01	0.16		5.72	24.77	0.65
110	1.00	Optimistic	.5		1.58	0.40			3.13			
61	1.00	Pessimistic	0	0.54	2.43	0.29			3.64			0.97
34	1.00	Optimistic	.5			0.23			12.44			0.77
**Mean**	0.64			0.40	1.03	0.20	0.16	0.22	7.40	7.81	5.76	0.43
**STD**	0.25			0.29	1.11	0.17	0.30	0.25	7.28	11.09	6.76	0.60

Supplementary Table 1. Parameter estimates for the selected model for each subject in experiment 1. Subjects are arranged from lowest to highest average free choice preference (column 2). Empty cells indicate that the corresponding parameters were not included in the winning model and were ignored for calculating summary statistics.

Experiment 2

Id	Free choice preference	Target policy	Q-value initial-ization	Pessimist weight (β)	Free choice bonus (ρ)	Learning rate (α)	Learning rate, t=1 (α)	Learning rate, t=2 (α)	Softmax ($1/\tau$)	Softmax t=1 ($1/\tau$)	Softmax t=2 ($1/\tau$)	Choice stickiness (κ)
1034	0.00	Pessimistic	0	0.03		0.15			8.44			
1103	0.20	Optimistic	0			0.03			8.14			0.09
1006	0.22	Optimistic	0			0.01			5.12			0.39
1036	0.23	Optimistic	0				0.47	0.11	2.99			0.44
1037	0.24	Pessimistic	.5	0.60		0.47			4.24			
1035	0.28	Optimistic	0			0.32			4.06			0.34
1025	0.31	Optimistic	0				0.03	0.47	6.65			0.10
1018	0.33	Optimistic	.5			0.05			21.57			0.07
1005	0.33	Optimistic	.5			0.05			9.46			0.19
1010	0.33	Pessimistic	.5	0.01	0.66	0.24			6.48			0.19
1021	0.34	Optimistic	0			0.17			6.96			0.20
1032	0.39	Optimistic	0		4.18		0.00	0.76	0.45			4.45
1027	0.42	Optimistic	0				0.00	0.11	4.02			0.49
1020	0.46	Optimistic	0		1.59		0.01	0.94	0.73			1.83
1017	0.48	Optimistic	0				0.25	0.98	2.47			0.84
1015	0.48	Optimistic	0			0.20			3.80			0.22
1029	0.48	Optimistic	.5			0.01			11.15			
1104	0.48	Optimistic	0			0.02			14.75			0.08
1028	0.48	Optimistic	.5		0.28	0.05			4.31			
1022	0.51	Pessimistic	0	0.17	1.45	0.09			1.82			0.73
1008	0.54	Optimistic	0		2.28	0.13				0.12	1.12	
1009	0.55	Pessimistic	.5	0.35	0.65	0.13			5.70			0.19
1013	0.57	Optimistic	.5				0.01	0.19		0.68	4.60	1.12
1019	0.59	Optimistic	.5		0.33		0.38	0.03	7.45			0.11
1014	0.60	Optimistic	.5		0.38	0.63			1.83			
1007	0.61	Optimistic	0		0.72	0.39			0.96			
1012	0.61	Optimistic	0		0.62		0.22	0.72	2.66			0.29
1102	0.64	Optimistic	0				0.00	0.64	3.04			0.51
1101	0.68	Optimistic	.5		0.40	0.05			6.18			0.15
1023	0.80	Optimistic	.5		0.47		0.05	0.58	3.13			0.25
1031	0.84	Optimistic	.5		1.91		0.42	0.00	15.95			
1033	0.88	Optimistic	0		2.86	0.62				0.67	1.48	0.65
1016	0.92	Optimistic	0		1.26		0.57	0.02	2.78			0.35
1011	0.94	Optimistic	0		2.04		0.03	0.48	1.28			0.87
1030	1.00	Optimistic	0		5.38		0.10	0.75	2.40			1.77
1024	1.00	Optimistic	.5		3.17		0.02	0.21	1.35			1.16
**Mean**	0.52			0.23	1.61	0.19	0.16	0.44	5.52	0.49	2.40	0.65
**STD**	0.24			0.25	1.43	0.20	0.20	0.34	4.74	0.32	1.92	0.88

Supplementary Table 2. Parameter estimates for the selected model for each subject in experiment 2. Subjects are arranged from lowest to highest average free choice preference (column 2). Empty cells indicate that the corresponding parameter were not included in the winning model and were ignored for calculating summary statistics.

Reviewer #2:

In this article, the authors aimed to identify the features triggering the well-known preference for free choices over forced choices. The authors replicated this effect in a 2-step task with two arms at step 1 –one leading deterministically to a free choice at the second step, the other to a forced option. The second-step terminal cues were probabilistically related to a binary outcome with the probability P of getting a reward being the same for both the best free choice outcome and for the forced cue outcome. Importantly, the experiment was divided into two phases: a training phase allowing participants to learn the terminal option-reward contingencies followed by a testing phase measuring free choice preference above and beyond learning. The main results are that participants prefer free choices after learning despite the risk of making a bad decision in step 2 free choice –even with a risk is maximised with P = 1 (Experiment 1). This result rules out that exploration would explain free choice preferences. Yet, this preference is reversed when the expected value of the best free choice option is much lower than the expected value of the forced option (Experiment 2). The authors offer a model-based mechanism posing that free choice preferences emerges from a bias placed on each free choice reward in a reinforcement learning model. Finally, they claim that free choice preference depends on personal control experience when the task is controllable.

Overall, this paper is well-written and I found it very interesting and exciting as free choice and more globally intrinsic motivation seem to be ubiquitous in everyday life and yet remains very poorly understood. It is also a growing topic of interest in the computational and cognitive neuroscience community. Here, the authors carefully designed experiments to test for various factors potentially influencing free choice preference. Moreover, they offered a mechanistic account for free choice preference using a model-based approach that is carefully and purposefully driven, ruling out many potential motivations for free choice preference. Most of the results are informative and neat.

My main concern is that the paper seems to fail identify what is behind free choice preference. Although the authors rule out many potential explanations through experiment 1 and 2, the data seem to rule out their proposed explanation in experiment 3 –personal control. See below for more details.

We thank the reviewer for the positive comments and questions as well as constructive criticisms of our manuscript. We address each point in detail below and provide new data and several new analyses and expanded arguments that we believe greatly strengthen our revision.

As a brief precis, we note that some of the alternatives that we rule out as general explanations of choice preference do not rely on outcomes being associated with self-determined actions. For example, exploration or Q-learning could be accomplished by instructed or forced sampling or even passive observation. Such off-policy strategies ensure sufficient sampling of the environment so that when actions are taken, there is a better chance of maximizing reward. Data from our experiments 1 and 2 show that on average human subjects continue to prefer choice despite potential loss (experiment 1, deterministic block P = 1) or actual loss (experiment 2, when P_forced > P_free). Moreover, even though subjects selected the suboptimal 1st-stage option, they still chose the better rewarded 2nd-stage target. These data are already consistent with the hypothesis that subjects prefer to exercise personal control. That is, they express behavior consistent with attributing value to being able to choose their course of action. We designed experiment 3 to address two further possibilities: i) that subjects instead preferred action variability, and ii) choice seeking was linked to personal control over eventual rewards (action-state-reward sequence). In experiments 1 and 2 it is possible that subjects preferred choice because the forced option always led to the same obligatory action (no variability). Regarding (ii), experiments 1 and 2 actions always led to a fixed state (unique fractal), and 2nd-stage choices may have been implemented independently of the motivation underlying 1st-stage choice. That is, subjects might have selected the free option based simply on the opportunity to pick a fractal of their choice (motivated purely by proximal control). Alternatively, they may select the free option based on the expectation that proximal control will lead to distal consequences (i.e., the subsequent state and

eventually the extrinsic outcome). These are not necessarily exclusive (see detailed responses below to questions about bias and potential variability), but they are both consistent with our personal control hypothesis. Data from experiment 3 demonstrates that subjects preferred choice even when action variability was maximal for the forced option (i, mentioned at L 255 of original manuscript), and that despite equal reward probabilities subjects are sensitive to outcomes from self-determined actions (ii, that is, when they are consistent with the expected state outcome of their own action).

Thus, we think that experiment 3 adds further support for our personal control hypothesis by linking the 1st-stage choice with the 2nd-stage consequences. We appreciate that we may not have been sufficiently clear, and as detailed below, the reviewer's comments below have helped us to significantly clarify and improve our manuscript.

1) Experiment 3 aims to show that free choice preference is due to personal control or at least that it is influenced by personal control. To test this idea, stage 2 choice controllability (i.e. how likely it is that the chosen option is indeed selected) is altered, with the prediction that free choice preference should decline as controllability decreases.

1a) Yet, this may not to be the case in the data (see Figure 5B and mostly figure S3B that would deserve to be kept in the main text rather than to be hidden in the supplementary material). In fact, the crucial test for the effect of controllability on free choice preference is not reported. Therefore, the following claim lack of statistical support and is currently not appropriate: "Increasing the incoherence of the 2nd-stage actions progressively reduced choice preference (block 2 and 3: 67% and 64% in favor of free respectively).". Statistics should be reported and interpreted with the corresponding claim.

We originally placed this figure in the supplementary results to move directly to examining the influence of the previous trial. The original figure showed the choice preference conditional on the previous choice, which indeed leaves implicit the marginal average choice preference. We certainly appreciate that this is not obvious and could benefit from a figure. As requested, we have integrated Fig.S3 as main Fig. 6 in the revised manuscript. We also added the requested statistic, which shows that the decline in choice preference with increasing incoherence is not statistically significant. We modified the sentence highlighted by the reviewer to "Increasing incoherence decreased 1st-stage choice preference by 2.4 and 5.4 points in blocks 2 and 3 respectively compared to block 1 (full controllability; Fig. 6B). This decrease was not significant (estimated trend = -1.55, 95% CI = [-4.45, 1.36], p = 0.298; see Supplementary Fig. 3 for individual subjects)." (p.18 of revision).

However, this lack of significance does not necessarily lead to the conclusion that manipulating controllability does not support our personal control hypothesis. In sequential decisions, there are many possible ways that past task outcomes can influence current and future decisions. For example, interactions with previous outcomes are useful to test whether subjects take the transition probabilities into account in other two-stage tasks (e.g., Daw et al., Neuron, 2011), which directly motivated our analyses and the way we originally presented it. Indeed, choice preference in the face of diminished control could arise because subjects simply preferred making a choice, irrespective of the outcomes (when they are equal). What we showed by analyzing 2nd-stage stay behavior is that despite equal reward probabilities subjects are sensitive to outcomes from self-determined actions (action-state-reward sequence when it is consistent with the expected state outcome of their own action). This occurs together with a strong tendency to repeat selection of the free option at the 1st-stage, linking 1st-stage choice with the 2nd-stage consequences.

Nevertheless, we did seek to provide additional evidence that our controllability manipulation modulates subject behavior at the first stage. To do so we examined the reaction times (RT) of individual subjects and found that, RTs are speeded in experiment 3 (when the free option is selected as well as a function of increasing incoherence). These RT results are now available in the new Supplementary Figure 2 (reproduced below) as well as p.6 (experiment 1), p.9 (experiment 2) and p.18 (experiment 3) of the revised manuscript. We believe that this RT effect provides additional evidence that controllability modulates subject behavior, which may be of interest to readers.

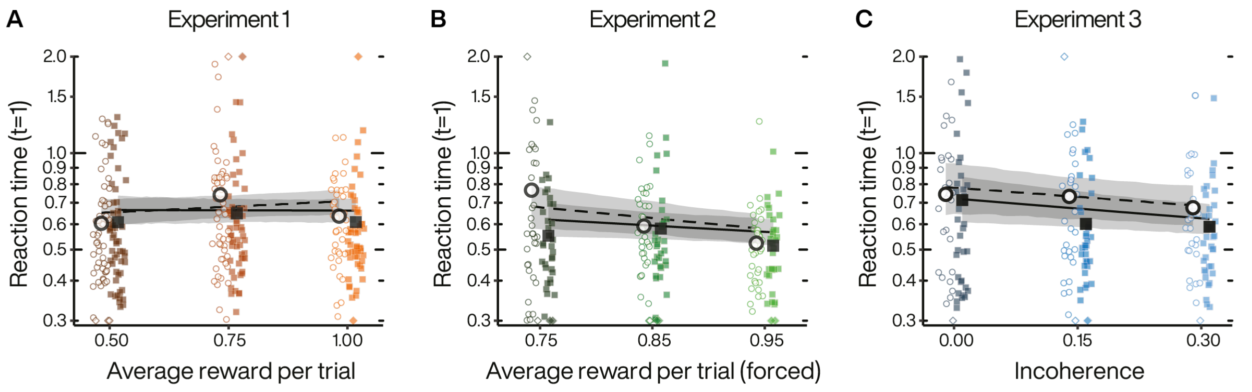

Supplementary Figure 2. First-stage reaction times for the different conditions in each experiment. **A.** Reaction times as a function of extrinsic reward probabilities in experiment 1. Lines indicate the estimated median reaction times with 95% credible intervals for reaction times corresponding to *free* (solid) and *forced* (dashed) selections. Black squares and circles represent the mean of individual subject median reaction times for *free* and *forced* selections respectively. Colored symbols represent individual subject medians. Overall estimated trend = 0.054, 95% CI = [-0.062, 0.175] (p = 0.370). Estimated trend forced = 0.117, 95% CI = [-0.025, 0.259] (p = 0.109). Estimated trend free = -0.009, 95% CI = [-0.141, 0.116] (p = 0.890). **B.** Same as A but as a function of extrinsic reward probabilities of *forced* trials in experiment 2. Overall estimated trend = -0.367, p5% CI = [-0.694, -0.024] (p = 0.020). Estimated trend forced = -0.464, 95% CI = [-0.884, -0.076] (p = 0.016). Estimated trend free = -0.268, 95% CI = [-0.651, 0.091] (p = 0.128). **C.** Same as A and B but as a function of incoherence in experiment 3. Overall estimated trend = -0.279, 95% CI = [-0.570, 0] (p = 0.040). Estimated trend forced = -0.309, 95% CI = [-0.660, 0.0329] (p = 0.060). Estimated trend free = -0.250, 95% CI = [-0.548, 0.024] (p = 0.063).

1b) Similarly, the following statement can remain as such only if statistics indeed support an overall decline in free choice preference as incoherence increases. It should be removed otherwise. LL 261-262 "We found that *the decline in choice preference* depended on the 1st-stage choice on the previous trial." I found the other arguments supporting the need for personal control explanation of free choice preference rather vague:

We have added the requested statistics (p.18):

"We found that choice preference depended on the choice made on the previous trial (Fig. 7A), with a significant main effect of previous 1st-stage choice (χ^2 = 451, p < 0.001) as well as a significant interaction between the previous 1st-stage choice (*free* or *forced*) and the degree of incoherence (χ^2 = 11.5, p < 0.001). Specifically, the frequency of *free* selections after a *forced* selection decreased significantly with incoherence (estimated trend = -2.68, 95% CI = [-4.92, -0.435], z = -2.34, p = 0.019), whereas the frequency of repeating a previous *free* selection did not change significantly with incoherence (estimated slope = -0.078, 95% CI = [-2.23, 2.07], z = -0.071, p = 0.943)."

We address the vagueness of our subsequent arguments in the following comment.

2) I do not see how to interpret that "as incoherence increased, subjects tended to stay more with the forced option, while maintaining a preference to repeat free selections" and I am not sure that it is necessary to show that the motivation for personal control is what drives free choice preference. I suggest to better explain how important it is for the demonstration or to move it in the supplementary material.

We thank the reviewer for her/his comment, and we endeavored in our new manuscript version to better explain this aspect of the manuscript. This point was also raised by reviewer 3, so we think it is important to make the logic behind this reasoning clearer. Therefore, we have rewritten parts of the results and discussion section regarding the controllability experiment and the personal control hypothesis. We have tried to better connect the context-dependent repetition observed in experiment 3 (that is, following incoherent trials, participants repeat the initial selection regardless of choice outcome) with the broader psychology of control maintenance and threat-compensation strategies. In a nutshell, we suggest that repetition is a response to certain features of Experiment 3 (the target swaps), which reduce control, a reduction that participants compensate for by repeating the initial selection. We hope that these new developments make our reasoning clearer.

Below is extended argumentation to clarify further about why we believe personal control is associated to free choice preference:

In Experiment 3, we observe that participants ignore the past reward when the terminal state (the selected target) is incoherent and thus more difficult to attribute to one's own action. We suggest that discounting the outcomes of events that are not caused by one's own choices, even though a choice is made, is a way for participants to maintain the consistence of their beliefs in their instrumental capacities – that is, voluntary actions are generally followed by consequences that can be reliably predicted. What our data suggest is that this belief may be a default, and that the free-choice preference observed in our experiments instantiates this default belief.

As we mention in the Discussion, this default control belief has been described in the literature under many terms and concepts – whether self-efficacy (Bandura), sense of agency (Haggard), perceived behavioral control (Ajzen) or internal locus of control (Rotter). What has been widely documented is that when this control belief is threatened or challenged, subjects develop various strategies to restore it (Brehm and Brehm, 1981). Note that a variety of compensatory strategies for reduced control has been documented in humans: for example, individuals experiencing loss of control are more likely to use placebo buttons—to repeatedly press buttons that have no real effect—or to see images in noise or to perceive illusory correlations, etc. We suggest that discounting of choice outcomes is an occurrence of such a compensatory strategy: participants discount choice outcomes to compensate for the reduction in control induced by target swaps, consistent with the broader psychology of control maintenance and threat-compensation literature (see Landau, Kay & Whitson, 2015, Psych Bull, for a review).

Repeating the incoherent free trials would thus simply be the corollary of discounting the outcomes of this trial. Note that this repetition is more than simple perseveration because it occurs specifically when the subject performs a voluntary action (free choice) and witnesses an incoherence between that voluntary action and its outcome. In this sense, it is reminiscent of the "try-and-try again" effect already observed in similar reinforcement learning studies measuring individuals' sense of agency in sequences of trials alternating free and forced choice (di Costa et al., 2018) or the "choice streaks" exhibited by participants when making decisions under uncertainty (Alméras et al., 2022). Thus, when the situation is uncertain, or the feedback from their action is questionable or contradicts what they believe, participants try again and repeat the same choice until they feel they have gathered enough evidence to make an informed decision and change or not change their mind (Rouault et al., 2022). Similarly, the repetition of choices in Experiment 3 suggests that participants are reluctant to update their belief about their personal control and repeat the same action to test it again.

Note that we do not claim that this compensatory strategy, which results in the repetition of incoherent trials, is consciously implemented by the participants. In this sense, we recognize that some of our formulations in the manuscript may have been misleading, or at best confusing. We have therefore deleted or reworded all occurrences suggesting a deliberate or conscious strategy:

Results section, p.19:
"The sustained repetition of free selections across the different levels of incoherence suggests that subjects <u>may have been acting as if to maintain control over the task</u> through self-determined 2nd-stage choices."

Results section, p.20:

"Finally, an alternative strategy could rely on maintaining control in situations where control is threatened by adverse events (e.g., action-outcome incoherence). Subjects would thus ignore any choice outcome from trials with reduced controllability (incoherent trials) and repeat their initial choice selection (Fig. 7B, fourth panel)."

"Results of the stay behavior during 2nd-stage choice following free selection suggests that subjects consider the source of reward when choosing between the different fractal targets (Fig. 7C)."

"We suggest that this strategy reflects an attempt to maintain a consistent level of control over the environment at the expense of the task goal of maximizing reward intake."

Discussion section, pp.28:
"Thus, subjects appeared to ignore information about action-state-reward contingencies that was externally derived, and instead appeared to double down by repeating their past choice as if to maintain some control over the task. This behavior is consistent with many observations suggesting that when individuals experience situations that threaten or reduce their personal control, they implement compensatory strategies to restore their control to its baseline level(54,55)."

We also made the following changes to the abstract, Introduction, Results, and Discussion sections:

Abstract:
"When deciding between options that do or do not lead to future choices, humans often choose to choose. We studied choice seeking by asking subjects to decide between a choice opportunity or performing a computer-selected action. Subjects preferred choice when these options were equally rewarded, even deterministically, and were willing to trade extrinsic rewards for the opportunity to choose. We explained individual variability in choice seeking using reinforcement learning models incorporating risk sensitivity and overvaluation of rewards obtained through choice. Degrading task controllability diminished choice preference following forced actions, although willingness to repeat selection of choice opportunities remained unchanged. In choices following these repeats, subjects were sensitive to rewards following freely chosen actions, but ignored outcomes associated with reduced controllability, in a manner consistent with a desire to maintain personal control over outcomes. Choice seeking appears to reflect the intrinsic need for personal control, which competes with extrinsic reward properties and external information to motivate behavior."

Introduction, p.4:
"Finally, in the third experiment, we sought to test whether choice preference was motivated by personal control beliefs. We manipulated the controllability of the task and found that subjects' willingness to repeat a free choice was not affected by the lack of objective controllability over reward outcome. Importantly, subjects were sensitive to past rewards only in controllable trials, where state outcomes could be attributed to self-determined choice. In uncontrollable trials, however, subjects ignored reward outcomes and repeated their initial choice. We suggest that choice repetition in the face of uncontrollability reflects a strategy to compensate for reduced control over the environment, consistent with the broader psychology of control maintenance. Together, our results support the hypothesis that human preference for choice opportunities derives from the intrinsic motivation for personal control."

Discussion, p.28:
"Interestingly, when subjects selected the free option, the subsequent choice was sensitive to the past reward when the terminal state (the selected target) was coherent, and the reward could therefore be attributed to the subject's action. In contrast, subjects' choices were insensitive to the previous outcome when the terminal state was incoherent. Thus, subjects appeared to ignore information about action-state-reward contingencies that was externally derived, and instead appeared to double down by repeating their past choice as if they sought to maintain some control over the task. This behavior is consistent with many observations suggesting that when individuals experience situations that threaten or reduce their personal control, they implement compensatory strategies to restore their control to its baseline level(54,55). A variety of compensatory strategies for reduced control has been documented in humans. Thus, individuals experiencing loss of control

<u>are more likely to use placebo buttons–to repeatedly press buttons that have no real effect–to see images in noise or to perceive correlations when there are none (Whitson and Galinsky, 2008). We suggest that discounting of choice outcomes in incoherent trials, where personal control is threatened by unpredictable target swaps, is an occurrence of such a compensatory strategy. When faced with uncontrollability, participants would ignore any choice outcome and repeat their initial choice to compensate for the reduction in control, consistent with the broader psychology of control maintenance and threat-compensation literature (see Landau, Kay & Whitson, 2015, for a review).</u>

<u>Repetition of the initial choice in incoherent trials is more than mere perseveration (i.e., context-independent repetition) because it occurs specifically when the subject performs a voluntary (free) choice and witnesses an incoherence between that choice and its outcome. In this sense, it is reminiscent of the "try-and-try again" effect already observed in similar reinforcement-learning studies measuring individuals' sense of agency in sequences alternating free and forced choice trials (di Costa et al., 2018) or the "choice streaks" exhibited by participants when making decisions under uncertainty (Alméras et al., 2022). Thus, when the situation is uncertain, or the feedback from their action is questionable or contradicts what they believe, participants try again and repeat the same choice until they have gathered enough evidence to make an informed decision and change or not change their mind (Rouault et al., 2022). Similarly, the repetition of choices in Experiment 3 suggests that participants are reluctant to update their belief about their personal control and repeat the same action to test it again.</u>

<u>Finally, it should be noted that</u> this strategy <u>of ignoring choice outcomes from uncontrollable contexts</u> is at odds with a pure model-based strategy(41), where an agent could exploit information about action-state-reward contingencies whether it derived from their own choices (<u>controllable context</u>) or <u>from the environment or another agent</u> (<u>uncontrollable context</u>) (…)"

One final point that we think is important: we argue that repetition is a response to certain features of experiment 3 that reduce control (the target swaps), which participants compensate for by repeating the initial selection. Such compensation is not necessarily irrational, as the participants were not given any specific information about the target swaps. In such a context of uncertainty, they therefore have no particular reason to update their beliefs about their control abilities but would do better to reject the incoherent trial and "try again". This observation raises the interesting question of how much disconfirming evidence is needed for subjects to update their control beliefs, with some evidence suggesting that it takes more disconfirming evidence to change beliefs in controllable contexts than in non-controllable contexts (Weiss, Chambon et al., 2020, Nat Comms). One prediction that can be drawn for the present study is that the extent to which people repeat incoherent free trials is a function of the strength of their control belief: the more they believe they are in control of the task environment, the more they will need to repeat (i.e., accumulate disconfirming evidence) in order to update this belief. We believe this question can be addressed in future follow-ups directly testing associations between belief in personal control and the size of repetition sequences.

Added references:
Landau, M. J., Kay, A. C., & Whitson, J. A. (2015). Compensatory control and the appeal of a structured world. Psychological bulletin, 141(3), 694.
Whitson, J. A., & Galinsky, A. D. (2008). Lacking control increases illusory pattern perception. Science, 322(5898), 115-117.
Di Costa, S., Théro, H., Chambon, V., & Haggard, P. (2018). Try and try again: Post-error boost of an implicit measure of agency. Quarterly Journal of Experimental Psychology, 71(7), 1584-1595.
Alméras, C., Chambon, V., & Wyart, V. (2022). Competing cognitive pressures on human exploration in the absence of tradeoff with exploitation. PsyArXiv, doi:10.31234/osf.io/9qpuz.
Rouault, M., Weiss, A., Lee, J. K., Drugowitsch, J., Chambon, V., & Wyart, V. (2022). Controllability boosts neural and cognitive signatures of changes-of-mind in uncertain environments. ELife, 11, e75038.

3) The principal remaining argument to support that free choice preference depends on choice controllability is rather indirect. For all controllability levels and at stage 2, participants were sensitive to the previous reward when the selected option was the chosen

one (i.e. if the previous choice was coherent) but were not so when the selected option was not the chosen one (i.e. if the previous choice was incoherent).

We appreciate the opportunity to clarify our evidence and arguments. We address the reviewer's remark about the link between free choice preference and controllability in our response to the points that follow.

3a) Comparing statistically the incoherent stay proportion rewarded versus unrewarded would be required for this pattern to be fully demonstrated (as the stay proportion for coherent trials maybe lower than the unrewarded one). It should not be significantly different.

The reviewer is correct that one prediction for the personal control model is that the incoherent stay proportion does not differ between rewarded and unrewarded previous trials. This is indeed what we found. We mentioned briefly in the original legend that we only displayed significant comparisons in the figure, but we appreciate that readers may want to see all the effect sizes and associated p-values. We have therefore added these for all comparisons in the figure legend (Fig 7C in revised manuscript).

3b) Even then, I do not quite see how it proves that free choice preferences (measured at stage 1) depends on personal control. Perhaps it is because of the bonus placed on the reward in the learning model. Yet, a model with a choice bias may equally capture free choice preference (see below for more details on that point).

We believe we have addressed the first concern in our detailed response to this reviewer's point #2 above.

We treat the reviewer's point about choice bias in our detailed response to their point #5 below.

Without necessary evidence, abstract, results and discussion sections would need to be rephrased. I have other less problematic concerns and some suggestions which I hope will help improve this article.

We appreciate the reviewer previous comments and constructive criticisms about our results and interpretations for experiment 3. We hope that our new analyses and clarifications (some of which also appear in detailed responses below) have convinced her/him that subject behavior reflect an attempt to maintain control which is a key aspect of the preference for free choice.

4) The authors interpret the observed consistent free choice preferences over different reward probability values (.5,.75, 1) as evidence ruling out curiosity, exploration, variety-seeking and selection-based explanations. While I can see why it rules out curiosity and exploration, I am not sure regarding the other alternative explanations at least as described in the introduction.
4a) Regarding the selection-based explanations, it is not clear to me in the results section why cognitive dissonance would have reduced effect after learning when terminal reward probabilities are clearly different between each free choice options. It may be worth to unpack this explanation (as it is done in the discussion).

Thanks for pointing this out. Our claim about cognitive dissonance is based on prior work that measured the size of the dissonance effect while manipulating the relative value of two options, which shows that it is absent when relative value differences are small or equal (Izuma et al., PNAS 2010; Sharot et al, J Neurosci 2009). As the reviewer notes, this is mentioned in the Discussion, and we have now also unpacked this in the Results section as well (pp. 8).

4b) Regarding variety seeking, I guess that people may keep checking for changes in the

testing phase when the reward probability is <1 but not when it is 1 and that is why curiosity and exploration explanations would be ruled out when reward probabilities are very different. Yet, there is still potentially more variety following the free choice option (as one can choose the worst option) whatever the reward probabilities are. Can the authors clarify the link between the results and how this explanation is ruled out?

Yes, the deterministic reward condition is key for demonstrating that people can prefer choice in a condition where there is nothing left to explore or to be curious about. The reviewer raises the possibility that people might still be attracted to potential variety. We find it important to distinguish potential from expressed variety, which we considered explicitly. Subjects in the deterministic condition (P=1) of experiment 1 did not express variety since most subjects exclusively selected the best rewarded 2nd-stage target during the test phase. We acknowledge that subjects may prefer the free option in this condition because there is still potential variety that they rarely or never act on, and this preference could be captured via a bonus for the free option in experiments 1 and 2. Thus, in experiments 1 & 2, two different computational goals (maximizing potential variety versus maximizing personal control) can be achieved using the same algorithm (inflating free option subjective value). However, there is a feature of our experiment 3 that argues in favor of personal control over potential variety per se. In experiment 3 we modified the state structure of the task so that choosing the forced option led to the computer randomly selecting a fractal target in the 2nd-stage with equal probability. Of interest here is the full controllability condition, where insofar as potential variety can be equated with the variability of possible actions, then potential variety (and ultimately expressed variety) is maximal for the forced option. Yet subjects continue to prefer the free option, suggesting that, despite more variety following the forced option, subjects preferred to control their selection at the 2nd-stage. Imagine preferring a store that carries many brands of cereal over one that only sells your favorite, yet only ever buying your favorite brand. The idea of personal control is that rather than simply "keeping all options open", one prefers to "keep one's options open". This is, to our knowledge, the first time such a manipulation has been used to examine this aspect of choice preference. We thank the reviewer for proposing the idea of potential variety, which we now detail and use to sharpen and clarify text in the Discussion. There we make clear this distinction and restrict our claims to expressed as opposed to potential variety seeking when referring to experiments 1 and 2 (p.23). We also point out the implication for potential variety seeking when discussing experiment 3 (also p.23).

5) The model space the authors used missed a model that could equally capture free choice preference: a choice bias in the softmax. The current winning model assumes a bonus plugged to the reward which predicts that Pfree will be inflated with respect to Pforced due to the boost, and it would predict an increasing free choice preference as the number of learning trials increases. Yet, this is not testable in the current design precisely because the participants already learned when they had the choice between the step 2 free or forced choice. Currently, I do not see any data supporting the current best model. I suggest the authors to either add the model I suggest in the model space and stress that these are two possible mechanisms that cannot be distinguishable with the current experiment, or to run a new experiment to add the possibility for participants to make step-1 decisions at various learning stages.

We agree with the reviewer that choice preference could be generated by a bias added to the free state-action Q-value when a 1st-stage decision is made. While a bias towards the free option can generate choice preference, a key difference is that a free choice bonus can lead to altered action-value estimates since the inflated reward enters into subsequent value updates to modify Q-values. A bias, on the other hand, can generate free choice preference but will not enter directly into subsequent value updates (hence it will not directly affect action-value estimates). Importantly, rewards were probabilistically delivered (except for the one deterministic condition), meaning that the reward-prediction error learning rule could track local stochastic fluctuations in extrinsic rewards that could yield changes in free choice preference. So concretely, let a^1 represent the action that selects the free option, then a softmax policy biased towards free choice is:

$$\pi(s,a^1) = \frac{\exp([Q(s,a^1)+\text{bias}]/\tau)}{\exp([Q(s,a^1)+\text{bias}]/\tau)+\exp(Q(s,a^2)/\tau)}$$

Implementing a biased softmax policy is potentially useful when the bias on decision making is stable, as is the case for perceptual distractors (e.g., a flanker, see Sidarus, Chambon & Palminteri, 2019). Our initial decision to implement a free choice outcome bonus was directly motivated by prior experiments showing that cues associated with reward contingencies learned in a free choice context were overvalued compared to cues associated with the same reward contingencies learned in a forced choice context (Cockburn et al, Neuron 2014; Chambon et al., NHB 2020).

However, we appreciate that it is still interesting to know whether one model fits the subject data better. Therefore, we fit 24 additional models (one for each outcome bonus variant, replacing the outcome bonus with a policy bias) to each subject's data from experiments 1 & 2. First, we directly compared model evidence across all model pairs that were identical except that one was fit with a bonus and the other with a bias (2256 model pairs). This yields a distribution of differences (ΔBIC) with a mean = -7.76. This can be interpreted as an approximate Bayes factor that is strongly in favor of the outcome bonus model (2,3). We next examined the winning model for each subject. For 72% (68/94) of subjects the winning model was the same model that was selected before including the models with bias. Finally, we focused specifically on subjects where the winning model included a bonus or a bias (n = 63). We found that the best models for 59% (37/63) of subjects maintained a free choice outcome bonus when this parameter was originally included in the winning model, while for 30% (19/63) of subjects the best model switched from including an outcome bonus to including a bias instead. 11% (7/63) of subjects who did not have an outcome bonus originally ended up retaining a bias in the expanded model space. This indicates that the outcome bonus model fits best for most subjects. However, the preceding breakdown was based on ΔBIC of any magnitude and does not capture the strength of evidence. Therefore, we plot the ΔBIC (bonus – bias, lower is better) between the winning model and the next best model with a bonus or a bias (Supplementary Figure 7). This ΔBIC distribution has a mean = -13.8, which is strongly in favor of our original outcome bonus model. Moreover, there were 24/63 subjects where $|\Delta BIC|>20$ *(mean = 41.2), which constitutes very strong evidence in favor of the bonus model, versus 5/63 with the same strength of evidence in favor of the biased softmax policy (mean = 17.0). Taken together, these results indicate that a free choice outcome bonus fits the data better than a softmax bias, suggesting that most subjects update free and forced option values rather than applying a static bias. We have included the figure below and elements of the preceding response in the supplementary materials (Supplementary Figure 7 and associated text, pp.3-4 of Supporting information), and text in the results (pp. 14-15).*

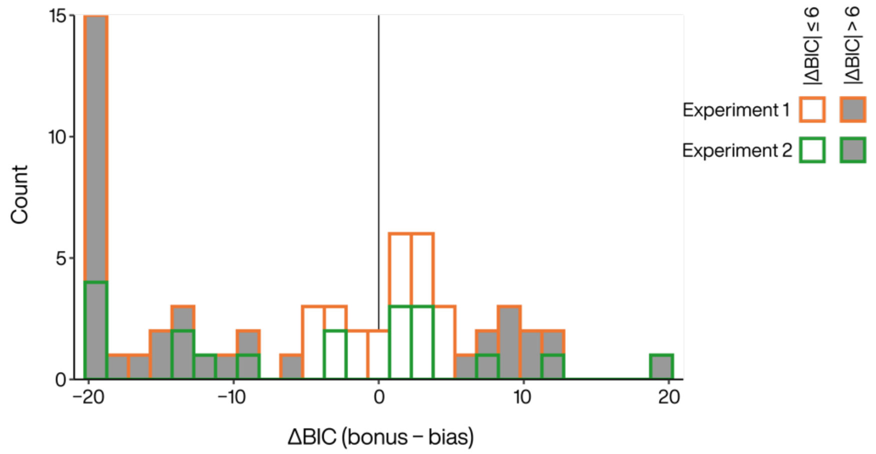

Supplementary Figure 7. Strength of evidence supporting free choice bonus model versus biased softmax policy model. Values along the abscissa represent the difference in BIC scores between the winning model and the next best model with a bonus or a bias for all subjects where the winning model included one of these parameters (*n* = 63). Differences less than -20 are included in the leftmost bin (*n* = 14) and differences greater than 20 are included in the rightmost bin (*n* = 1). Shaded bars indicate data where $|\Delta BIC| > \mathbf{6}$, corresponding to strong evidence in favor of the bonus model (negative values) or the bias model (positive values).

6) Here is another alternative model I suggest to add to the model space. The current best model includes a bonus placed on rewards following a free choice. If it actually predicts an increasing free choice preference, and if learning still occurs in the testing phase as suggested LL 184-188 pp 10-11, then it would predict an increasing free choice preference over trials in the testing phase. Yet, free choice preferences are constant (as can be observed in Figures 2C middle and right and Figure 3C left). I suggest to decrease the bonus strength linearly or exponentially for example as follows: rho_0 + rho_1 * trial number, or something like : rho_0 .* [rho_1 .^(trial number)]./rho_1 to account for the emergence of free choice preferences, with a reduced influence.

We agree with this reviewer that a free choice bonus could yield increasing free choice preference when Q-value differences are steadily increasing. It is important to point out that, since the bonus is an additive offset, Q-values will simply fluctuate around different mean values at steady state (i.e., equal to the expected value of the extrinsic reward plus the bonus for the free option Q-value). We used learning rates that did not change across trials, so in a sense the models can learn throughout the session (dictated by the stochastic rewards that would drive prediction errors). However, the models will initially attain a steady state as new data overwhelms initial Q-values, and the degree of fluctuation around the steady state is largely dictated by the learning rate. In general, one would not expect free choice preference to change after this steady state is obtained, which is mostly what we observed. We illustrate this in the figure below using a reinforcement learning model with a static learning rate and bonus. Therefore, we do not believe that adding a decreasing learning rate is necessary to explain the choice preference we observe in the testing phase.

We can integrate this figure in the supplementary material section of the manuscript if the reviewer thinks it appropriate.

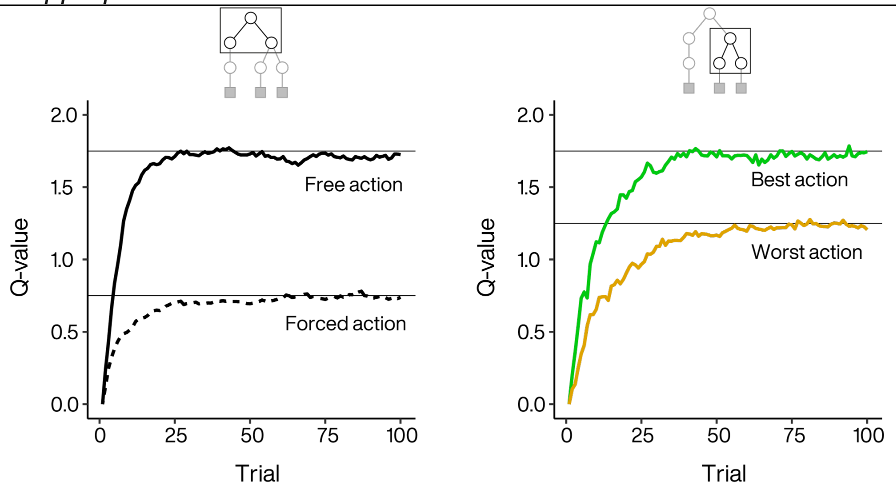

Figure R1. Simulation of a Q-learner with a free choice bonus. The left panel shows the average Q-values for state 1, where the model selects between the free and forced options. The right panel shows the average Q-values for state 3, where the model selects between two targets with different reward probabilities. Averages from 500 simulations, where the reward probabilities at the 2nd-stage were 0.75 and 0.25 for the best and worst actions (0.75 for forced action), learning rate = 0.35, and free choice bonus = 1. Thin horizontal lines indicate the expected values, which for the 2nd-stage are equal to 1.75 and 0.75 (expected rewards for each action plus free choice bonus). Q-values initialized at 0 for visualization.

7) It clearly appears that participant learned which free-choice option was the best in the Experiment 1 training phase. Yet, it is unclear what is learned: the actual probability or that one option is better than the other? In other words, were participants really learning P? If so, did they learned accurately P to the same extent for the forced and for the free choice condition or is P estimation inflated in free as predicted by the learning model with a boost placed on rewards? One way to check that would be to explicitly ask the participants for the probability of each cue to lead to a reward after the training phase. Was it measured in anyway?

This is a great question that was also posed by Reviewer 3. We actually collected data relevant to probability estimation and thank the reviewers for the opportunity to present it. We specifically asked the subjects in the experiment 2 to estimate the probability of extrinsic reward for each 2nd-stage fractal image. They did so by placing a cursor on a visual scale from 0 to 100 after completing each block of trials. We found that the subjects were relatively accurate at estimating the reward probabilities for fractals in both free and forced trials, with mild underestimation (overestimation) at higher (lower) reward probabilities (estimated trend forced = 0.749, 95% CI = [0.683, 0.816], t = 22.2, p < 0.0001; estimated trend free = 0.735, 95% CI = [0.640, 0.831], t = 15.2, p < 0.0001), which did not differ significantly between these trial types (estimated trend difference = 0.014, 95% CI = [-0.102, 0.13], t = 0.237, p =0.8124). This suggests that preference for the free option was not due to differential *distortion in estimating the* frequency *of rewards. We think this is novel information that we had not thought to examine before, and we thank the reviewer for making this idea clear to us. We have integrated the following figure into the main text (new Figure 4 in revision), with associated text (pp. 11-12).*

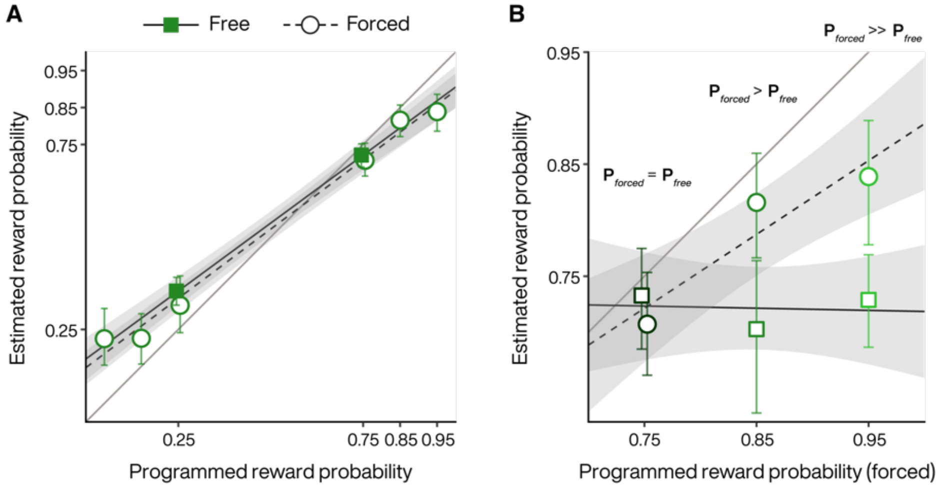

Figure 4. Subject estimates of extrinsic reward probabilities in experiment 2. **A.** Each circle (forced) or square (free) represents the average estimate of each 2nd-stage images experienced or viewed by the subjects across the three blocks. The estimates for fractals in the free option were averaged across the blocks but were similar both for the poorly rewarded targets (0.39, 0.35 and 0.32 for P_{forced} = 0.75, 0.85 and 0.95 respectively) and the highly rewarded targets (0.73, 0.70 and 0.73 for P_{forced} = 0.75, 0.85 and 0.95 respectively)). Black lines with shading indicate the estimated trends from a GLMM, with 95% CI. **B.** Same as in A but only for the highly rewarded targets (i.e., the most selected, see Fig. 3). Note that in contrast to A., the abscissa in B. refers to the maximal reward probability for the forced option. P_{free} did not vary significantly (estimated trend = -0.019, 95% CI = [-0.331, 0.293], p = 0.902), consistent with the maximal reward probability for the free option being constant at 0.75. Black lines with shading indicate the estimated trends from a GLMM, with 95% CI.

8) Are participants aware that the free choice corresponds to a 1-arm bandit (option 1 reward probability = P; option 2 reward probability = 1-P) and not to a 2-arm bandit (option 1 reward probability = P; option 2 reward probability = P')? A possibility is that participants may overestimate the worst outcome reward probability depending on their prior (e.g. neutral priors 0.5 placed on each Q value at trial 0) and given that they did not choose them a lot and therefore given that they did not update them a lot. As a consequence, the free choice expected-value maybe higher than the forced choice expected value. This could be tested by mean of model comparison (even when the objective reward probability was set to be 1). I think that it would require Q to be at 0.5.

Subjects were not informed about the actual reward probabilities and learned this knowledge from experience. Thus, as the reviewer points out, subjects may have overestimated the worst outcome reward probability. It is worth noting that the task design imposes that this overestimated probability must be greater than or equal to the best outcome reward probability in order to exceed or equal the expected value of the forced option. Say, for a true best outcome reward probability

P=0.75, the expected value for the forced option is 0.75 since the computer always selects the best target, and a subject would have to believe that the worst *outcome probability in the free option is ≥ 0.75. This is not the case, as illustrated in the new figure presented in the preceding response. There we see that subjects accurately estimate reward probability associated with fractals in the free option, even when they relatively rarely sampled the poorly rewarded fractal (P=0.25). Importantly, the average estimate for the worst rewarded fractal (mean = 0.35) is significantly below the 0.75 needed to match the expected value of the forced option (t = -20.1, p < 1e-6).*

We also asked subjects to estimate the reward probability for the fractals that were never chosen by the computer in the forced option (Figure 4A above, programmed reward probabilities for the forced option = 0.25, 0.15, 0.05). These probability estimations were not based on direct experience, and subjects appear to have inferred that it was 1-P from their experience in free trials, or alternatively this may be the consequence of a kind of counterfactual prior belief (Alméras C. et al, 2022).

We thank the reviewer for their detailed comment, and we have incorporated elements of our response into the revised manuscript (new Fig. 4, pp. 10-12):

"We also asked subjects in experiment 2 to estimate the extrinsic reward probabilities associated with each 2nd-stage fractal image. They did so by placing a cursor on a visual scale from 0 to 100 after completing the test phase of each condition. We found that the subjects were relatively accurate at estimating the reward probabilities for fractals in both *free* and *forced* trials (Fig. 4A), with mild underestimation (overestimation) at higher (lower) reward probabilities (estimated trend *forced* = 0.749, 95% CI = [0.683, 0.816], $t = 22.2$, $p < 0.001$; estimated trend *free* = 0.735, 95% CI = [0.640, 0.831], $t = 15.2$, $p < 0.001$), which did not differ significantly between these trial types (estimated trend difference = 0.014, 95% CI = [-0.102, 0.130], $t = 0.237$, $p = 0.812$). This suggests that preference for the *free* option was not due to differential distortion in estimating the frequency of rewards (Fig. 4B). In addition, subjects did not overestimate the worst reward probability sufficiently to explain their preference. Note that by design such an overestimated probability must be greater than or equal to the best outcome reward probability in order to exceed or equal the expected value of the *forced* option. Say, for a true best outcome reward probability P=0.75, the expected value for the *forced* option is 0.75 since the computer always selects the best target, and a subject would have to believe that the worst outcome probability in the *free* option is ≥ 0.75. This is not the case, and the average estimate for the worst rewarded fractal (mean = 0.35) is significantly below the 0.75 needed to match the expected value of the *forced* option ($t = -20.1$, $p < 0.001$). Finally, we also asked subjects to estimate the reward probability for the 2nd-stage fractals that were never chosen by the computer in the *forced* option (Figure 4A, putative programmed reward probabilities = 0.25, 0.15, 0.05). These probability estimations were not based on direct experience, and subjects appear to have inferred that it was 1-P from their experience in *free* trials, or alternatively this may be the consequence of a kind of counterfactual prior belief (34)."

We address the reviewer's question about Q-value initialization in the next response.

9) Why are Q initialised at 0? Does model comparison show that it is better than 0.5?

We thank the reviewer for posing this question, which was also raised by Reviewer 3. Q-value initialization is indeed an important hyperparameter. In the machine learning literature, Q-values are frequently initialized at 0 when other mechanisms are incorporated to promote exploration (ε-greedy or softmax decision rules (1)). Since we used the softmax decision rule we originally initialized the Q-values at 0 because, like initializing at 0.5, it indicates initial indifference between options (this decision rule considers only Q-value differences). However, Reviewers 2 and 3 raise the possibility that initializing at 0.5 might better represent the subjects' priors and therefore provide a better fit to their data. To address this, we fit the whole set of models using initial Q-values = 0.5 (4512 model fits). Briefly, the outcome of our analyses is that initial Q-values = 0 is overall superior to initial Q-values = 0.5, and that using the model with initial Q-values = 0.5 for those individual subjects where it provides a better fit does not change our conclusions.

First, we directly compared model evidence across all models with initial Q-values = 0 and 0.5, which yields a distribution of differences (ΔBIC) with a mean = -17.6. This can be interpreted as an approximate Bayes factor that is strongly in favor of initial Q-values = 0 (2,3). We next examined this more closely by comparing the best model selected for each subject under each initialization (Supplementary Figure 8, left panel). Again, the evidence is in favor of initial Q-values = 0, with a mean difference = -4.38, which can also be observed using Tjur's (4) R^2 to measure model fit, although the average difference in fit is relatively modest (Supplementary Figure 8, right panel). For 63% of subjects (59/94), the model with initial Q-values = 0 is selected (ΔBIC<0). In 40% of the cases (14/35) where the model with initial Q-values = 0.5 is selected (ΔBIC>0), the selected model was structurally identical to that selected for initial Q-values = 0 (i.e., same target policy, presence/absence of free choice bonus, etc.). In the remaining 21/35 subjects, 8 exhibited ΔBIC>6, which constitutes strong evidence (2) in favor of initial Q-values = 0.5. For these, we examined our main parameter of interest, the free choice bonus, and we found that this parameter was retained in every case where it was retained in our initial fits with initial Q-values = 0 (n=6). Finally, we compared the free choice bonus for all models where it was retained for both initial Q-values = 0 and 0.5 (n = 51) and found that the estimated bonus parameters were significantly correlated (r = 0.838, p < 1e-6).

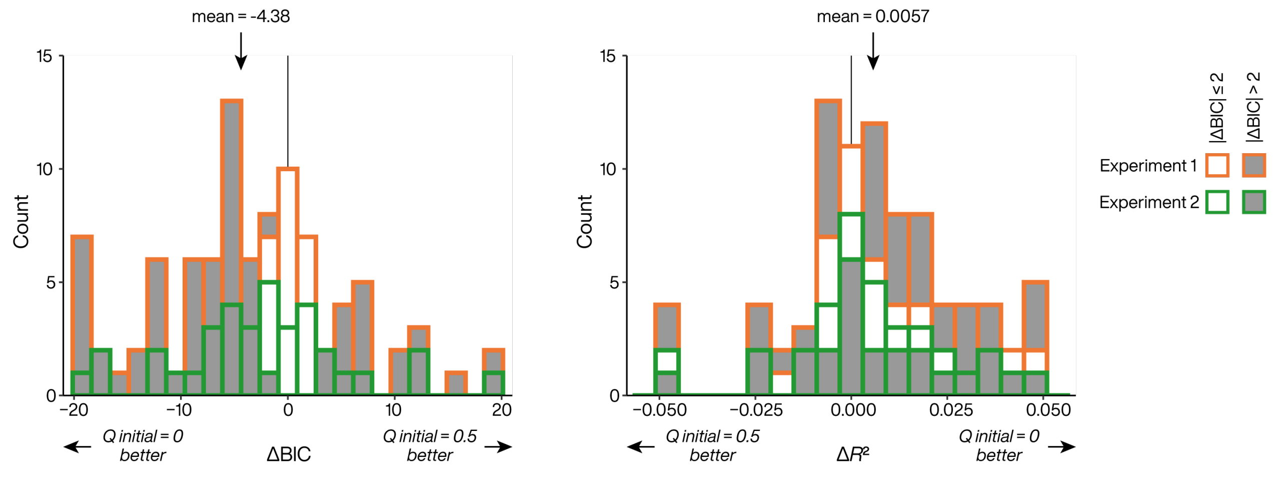

Supplementary Figure 8. Comparing models fit with initial Q-values equal to 0 or 0.5. The left panel shows **Δ**BIC scores (negative values favor initial Q-values = 0), and right panel shows delta Tjur's $\boldsymbol{R}^2$(positive values favor initial Q-values = 0).

Taken together, these analyses indicate that, under the conditions of our task, the evidence supports our original choice of initial Q-values = 0. However, some subjects were indeed better fit with initial Q-values = 0.5. We have therefore revised the manuscript to select the best Q-value initialization for each subject. This is detailed in the methods (pp. 37-38 in the main new manuscript and pp.4-5 of the new Supporting information). Also, new Figure 5 (originally Fig. 4), supplementary

Figure 6 (originally Sup. Fig. 2) and new Supplementary Figure 8 have been updated to reflect these minor changes, which do not alter any of our conclusions.

10) Results showing free choice preference are a weakened by the fact that the extrinsic rewards were actually not implemented. It may be that participants only prefer free choices over forced choices when the cost is very low. In the current experiment, a reward probability difference of 0.2 is enough to reverse preferences at the group level. Yet, if extrinsic rewards were real, a much lower difference (maybe no difference) would be enough to reverse the effect. Adding this condition would make the result stronger. Alternatively, I suggest the authors to stress that the results validity is limited by the lack of actual extrinsic rewards.

When the reviewer states "extrinsic rewards were actually not implemented" we take them to mean that subjects were not compensated as a direct function of the reward feedback earned in the task (i.e., each reward symbol did not lead to fixed monetary amount that was accumulated and paid out exactly at the end of the session). We agree that this is a limitation, and we have therefore modified the discussion to point out this limitation (p. 25):

*"*One limitation of our experiments is that subjects were not compensated as a direct function of the reward feedback earned in the task (i.e., each reward symbol did not lead to fixed monetary amount that was accumulated and paid out exactly at the end of the session). It is plausible that direct monetary feedback could lead to differences at the group and individual level. For example, increasing the direct monetary payoff associated with every successful action could be expected to eventually abolish choice preference for most if not all subjects (in the deterministic condition in experiment 1 a subject risks losing a potentially large payoff due to choosing incorrectly at the 2nd-stage). Understanding how direct reward feedback influences choice preference will be important for better understanding the role of intrinsic motivation for choice in real world decision making.*"*

11) I would not frame Experiment 2 as a titration. In this experiment, Pforced is either the same as Pfree, (Pforced=Pfree=.75), either larger (Pforced = .85 > Pfree), or much larger (Pforced = .95). The group average when Pforced =.85 is indeed around .5. Yet, the variance is very high and the distribution seems rather bimodal with a very few participants being indifferent between free choice and forced choice. I suggest the authors to frame it as a reversal: when the difference between Pforced and Pfree is 0.20 the vast majority of participants reversed their preference compared to when Pfroced=Pfree.

We agree with the reviewer's point about preference reversal and have removed the term "titration" in our revised manuscript. We also reinforce this point on p.9 of the revised manuscript.

12) Results regarding the free choice preference are convincing (e.g; Figure 2B). Yet, there is a lot of variance in the data. Do the authors have a clue about why some participants always prefer the forced choices? Are they the ones who learned faster in the training phase?

Indeed, many subjects did prefer the forced option for one or more experimental conditions, which contributed to the high variance in preference within and across conditions (this is particularly relevant in experiment 1, where the maximal expected free and forced option extrinsic rewards are

equal). This variability led us to explore the pessimistic target to capture different subject strategies. We believe that the idea that individual subjects differentially weight the best and worst outcomes is quite natural and goes some of the way towards explaining the observed inter-individual variability (Figure 5 in revision). Other subject-specific properties surely contribute, and we tested the reviewer's proposal that learning speed may be one of these. There was only one subject in experiment 1 (Figure 2B) that nearly exclusively preferred the forced option (Id# 108 in new Supplementary Fig. 1), although two other subjects in experiment 1 also preferred the forced option > 80% of the time (Id# 78, 48 in new Supplementary Fig. 1. Their learning rates were lower than the average (Supplementary Table 1). To test the reviewer's suggestion across all subjects in experiment 1, we correlated subjects' average choice preference with the 2nd-stage learning rates estimated using the RL models. This yielded a positive correlation that was not significant (r = 0.140, t = 1.022, p = 0.311). By contrast, we observed significant positive correlations with free choice bonus (r = 0.370, t = 2.94, p = 0.005) and the degree of best outcome weighting in the β-pessimistic target (r = 0.340, t = 2.69, p = 0.009). We have added these correlations on page 14, reproduced here for convenience:

"Since some subjects preferred the forced option for one or more experimental conditions (Supplementary Figure 1), we examined whether individual parameters from the fitted TDRL models were associated with choice preference. We did not find significant correlations of average choice preference with that 2nd-stage learning rates (r = 0.140, t = 1.02, p = 0.311), softmax inverse temperatures (r = 0.033, t = 0.245, p = 0.807) or tendencies to repeat 1st-stage choices (r = 0.200, t = 1.54, p = 0.128). We did find that the magnitude of the free choice bonus was significantly associated with increasing choice preference (r = 0.370, t = 2.94, p = 0.005, Fig. 5C). We also found a significant correlation with the relative weighting between the worst and best possible outcomes in the β-pessimistic target (r = 0.340, t = 2.69, p = 0.009), with increasing weighting of the best outcome ($\beta \rightarrow 1$, see Material and Methods) being associated with increasing average choice preference."

13) It is not really clear what tests were performed in the result section. I suggest to report an effect size and to specify the test (as it seems to be a likelihood-ratio test, I suppose that the chi2 value could be reported).

We have revised the manuscript to 1) be more explicit about what tests were used (pg. 40), 2) include effect measures (e.g., estimated means or trends) and their associated test statistics or confidence intervals throughout.

14) I found it hard to get information about the model comparison results. In particular, figure 4A is not so informative as the models differs in terms of free parameters number. It is unclear what is the winning model at the group level and by how much. Reporting a figure or a table would be informative to get a sense of the second-best model, etc. In fact, reporting each model frequency on top would also be informative.

Thanks for pointing out this missing information, which we have now added in the form of a supplementary figure and two supplementary tables. This new material highlights the selected model for each subject as well as the relative weight of evidence for all fitted models (New Sup. Fig. 5), and reports estimated parameters and summary statistics (New Sup. Table 1 and 2). We present them below for convenience.

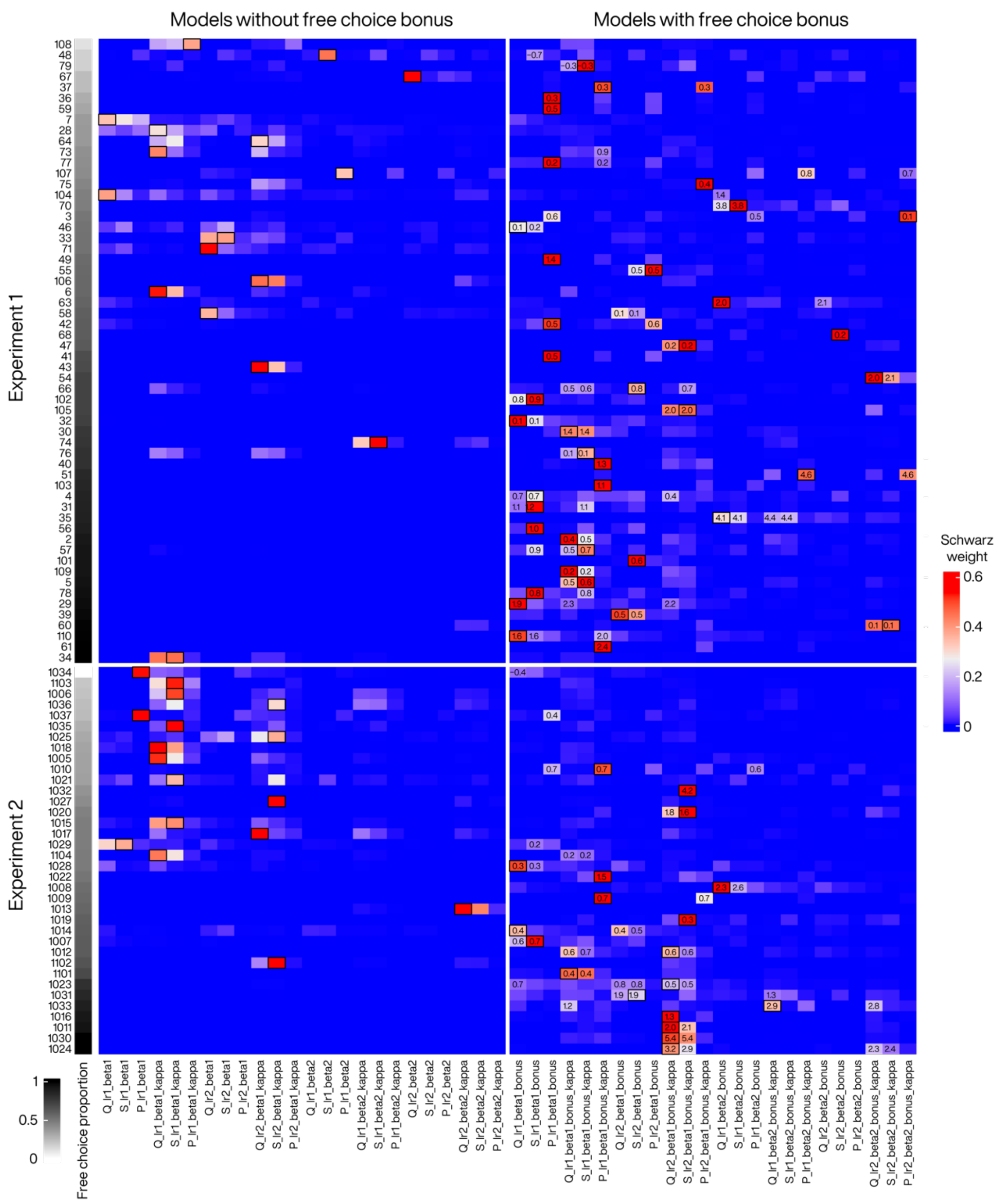

Supplementary Figure 5. Model selection for each subject in experiments 1 (top panels) and 2 (bottom panels). Each row represents the normalized BIC values (Schwarz weights) associated with each of the 48 models fitted to the data of each subject. These sum to one for each subject, and the selected model is highlighted by a black frame. The left panels contain models without a free choice bonus, whereas the right panels contain models with a free choice bonus. The column labels at the bottom give the model specification (target policy is given by the first letter, with Q = off policy maximum, S = SARSA, and P = β-pessimistic policy; lr = learning rate, which if followed by a 2 indicates a separate learning rate for the 1st- and 2nd-stage; beta = softmax temperature, which if followed by a 2 indicates a separate softmax temperature for the 1st- and 2nd-stage; kappa = choice stickiness; bonus = free choice bonus). Numbers in boxes give the free choice bonus (ρ) wherever the Schwarz weight > 0.10). Subjects are arranged from lowest to highest average free choice preference (top to bottom, for each experiment). See Supplementary Tables 1 and 2 for the other parameter estimates of the selected model for each subject.

Experiment 1

Id	Free choice preference	Target policy	Q-value initial-ization	Pessimist weight (β)	Free choice bonus (ρ)	Learning rate (α)	Learning rate, t=1 (α)	Learning rate, t=2 (α)	Softmax ($1/\tau$)	Softmax t=1 ($1/\tau$)	Softmax t=2 ($1/\tau$)	Choice stickiness (κ)
108	0.10	Pessimistic	0	0.75		0.08			7.37			0.20
79	0.17	Optimistic	0		-0.30	0.14			5.31			0.27
48	0.17	Optimistic	0			0.12				12.73	1.97	
37	0.24	Pessimistic	.5	0.03	0.32	0.18			9.91			0.16
67	0.24	Optimistic	0				0.03	0.14		28.99	5.59	
36	0.29	Pessimistic	.5	0.01	0.28	0.23			5.75			
59	0.32	Pessimistic	.5	0.01	0.47	0.19			6.77			
7	0.36	Optimistic	0			0.04			5.69			
28	0.37	Optimistic	0			0.14			9.27			0.06
64	0.37	Optimistic	0				0.23	0.01	12.65			0.14
73	0.40	Optimistic	0			0.07			5.23			0.23
77	0.41	Pessimistic	.5	0.38	0.22	0.09			14.51			
107	0.42	Pessimistic	0	0.02		0.10				0.47	6.81	
75	0.44	Pessimistic	0	0.40	0.43		0.03	0.30	5.68			0.40
104	0.47	Optimistic	0			0.08			4.50			
70	0.47	Optimistic	0		3.84	0.29				0.01	1.95	
3	0.50	Pessimistic	.5	0.75	0.13		0.02	0.05		29.36	10.65	0.00
46	0.51	Optimistic	0		0.12	0.04			6.77			
71	0.52	Optimistic	0				0.00	0.66	2.81			
33	0.52	Optimistic	0				0.01	0.07	5.06			
49	0.55	Pessimistic	0	0.43	1.37	0.59			5.04			
55	0.56	Pessimistic	.5	0.18	0.54		0.01	0.24	7.35			
106	0.56	Optimistic	0				0.01	0.26	8.66			0.17
6	0.56	Optimistic	0			0.03			6.33			0.14
63	0.58	Optimistic	0		2.00	0.48				0.16	1.62	
58	0.60	Optimistic	0				0.03	0.15	5.59			
42	0.60	Pessimistic	0	0.62	0.51	0.34			3.56			
68	0.60	Optimistic	0		0.24		0.04	0.17		13.66	2.28	
47	0.62	Optimistic	0		0.20		0.01	0.09	9.62			0.16
41	0.67	Pessimistic	.5	0.48	0.46	0.11			6.34			
43	0.68	Optimistic	.5				0.82	0.00	46.20			0.05
66	0.72	Optimistic	0		0.84		0.96	0.01	4.01			
54	0.72	Optimistic	0		2.04		0.01	0.59		0.57	2.45	2.22
102	0.72	Optimistic	0		0.95	0.04			2.09			
105	0.72	Optimistic	0		2.02		0.01	0.89	1.93			1.58
32	0.73	Optimistic	.5		0.06	0.06			22.55			
30	0.77	Optimistic	.5		1.42	0.13			2.48			-0.89
74	0.78	Optimistic	.5			0.24				1.14	8.49	1.64
76	0.80	Optimistic	0		0.11	0.07			11.89			0.10
40	0.80	Pessimistic	.5	0.34	1.32	0.11			1.75			0.44
51	0.84	Pessimistic	0	0.90	4.58	0.50				0.42	0.95	0.65
103	0.85	Pessimistic	0	0.60	1.06	0.13			2.54			0.38
4	0.86	Optimistic	0		0.75	0.13			3.02			
31	0.87	Optimistic	0		1.22	0.46			1.94			
35	0.88	Optimistic	0		4.11	0.58				0.52	1.64	
56	0.89	Optimistic	.5		1.00	0.38			2.72			
2	0.92	Optimistic	0		0.45	0.06			4.29			0.38
57	0.93	Optimistic	.5		0.67	0.05			3.98			0.27
109	0.93	Optimistic	.5		0.20	0.02			16.39			0.07
101	0.93	Optimistic	.5		0.61		0.01	0.09	10.53			
5	0.94	Optimistic	0		0.57	0.06			4.28			0.43
78	0.96	Optimistic	.5		0.83	0.47			5.23			
29	0.97	Optimistic	0		1.92	0.08			1.89			
39	0.98	Optimistic	.5		0.53		0.59	0.02	11.71			
60	0.99	Optimistic	.5		0.09		0.01	0.16		5.72	24.77	0.65
110	1.00	Optimistic	.5		1.58	0.40			3.13			
61	1.00	Pessimistic	0	0.54	2.43	0.29			3.64			0.97
34	1.00	Optimistic	.5			0.23			12.44			0.77
**Mean**	0.64			0.40	1.03	0.20	0.16	0.22	7.40	7.81	5.76	0.43
**STD**	0.25			0.29	1.11	0.17	0.30	0.25	7.28	11.09	6.76	0.60

Supplementary Table 1. Parameter estimates for the selected model for each subject in experiment 1. Subjects are arranged from lowest to highest average free choice preference (column 2). Empty cells indicate that the corresponding parameter was not included in the winning model and were ignored for calculating summary statistics.

Experiment 2

Id	Free choice preference	Target policy	Q-value initial-ization	Pessimist weight (β)	Free choice bonus (ρ)	Learning rate (α)	Learning rate, t=1 (α)	Learning rate, t=2 (α)	Softmax ($1/\tau$)	Softmax t=1 ($1/\tau$)	Softmax t=2 ($1/\tau$)	Choice stickiness (κ)
1034	0.00	Pessimistic	0	0.03		0.15			8.44			
1103	0.20	Optimistic	0			0.03			8.14			0.09
1006	0.22	Optimistic	0			0.01			5.12			0.39
1036	0.23	Optimistic	0				0.47	0.11	2.99			0.44
1037	0.24	Pessimistic	.5	0.60		0.47			4.24			
1035	0.28	Optimistic	0			0.32			4.06			0.34
1025	0.31	Optimistic	0				0.03	0.47	6.65			0.10
1018	0.33	Optimistic	.5			0.05			21.57			0.07
1005	0.33	Optimistic	.5			0.05			9.46			0.19
1010	0.33	Pessimistic	.5	0.01	0.66	0.24			6.48			0.19
1021	0.34	Optimistic	0			0.17			6.96			0.20
1032	0.39	Optimistic	0		4.18		0.00	0.76	0.45			4.45
1027	0.42	Optimistic	0				0.00	0.11	4.02			0.49
1020	0.46	Optimistic	0		1.59		0.01	0.94	0.73			1.83
1017	0.48	Optimistic	0				0.25	0.98	2.47			0.84
1015	0.48	Optimistic	0			0.20			3.80			0.22
1029	0.48	Optimistic	.5			0.01			11.15			
1104	0.48	Optimistic	0			0.02			14.75			0.08
1028	0.48	Optimistic	.5		0.28	0.05			4.31			
1022	0.51	Pessimistic	0	0.17	1.45	0.09			1.82			0.73
1008	0.54	Optimistic	0		2.28	0.13				0.12	1.12	
1009	0.55	Pessimistic	.5	0.35	0.65	0.13			5.70			0.19
1013	0.57	Optimistic	.5				0.01	0.19		0.68	4.60	1.12
1019	0.59	Optimistic	.5		0.33		0.38	0.03	7.45			0.11
1014	0.60	Optimistic	.5		0.38	0.63			1.83			
1007	0.61	Optimistic	0		0.72	0.39			0.96			
1012	0.61	Optimistic	0		0.62		0.22	0.72	2.66			0.29
1102	0.64	Optimistic	0				0.00	0.64	3.04			0.51
1101	0.68	Optimistic	.5		0.40	0.05			6.18			0.15
1023	0.80	Optimistic	.5		0.47		0.05	0.58	3.13			0.25
1031	0.84	Optimistic	.5		1.91		0.42	0.00	15.95			
1033	0.88	Optimistic	0		2.86	0.62				0.67	1.48	0.65
1016	0.92	Optimistic	0		1.26		0.57	0.02	2.78			0.35
1011	0.94	Optimistic	0		2.04		0.03	0.48	1.28			0.87
1030	1.00	Optimistic	0		5.38		0.10	0.75	2.40			1.77
1024	1.00	Optimistic	.5		3.17		0.02	0.21	1.35			1.16
**Mean**	0.52			0.23	1.61	0.19	0.16	0.44	5.52	0.49	2.40	0.65
**STD**	0.24			0.25	1.43	0.20	0.20	0.34	4.74	0.32	1.92	0.88

Supplementary Table 2. Parameter estimates for the selected model for each subject in experiment 2. Subjects are arranged from lowest to highest average free choice preference (column 2). Empty cells indicate that the corresponding parameter was not included in the winning model and were ignored for calculating summary statistics.

15) Lines 250-257 p 14: "Choice preference was high (70%) in block 1 when coherence was not altered, similar to block 1 from experiment 2 where extrinsic reward was equal between free and forced options". Can the authors report some statistics to support that claim?

We rewrote the paragraph including this sentence to provide the requested information (p.16 of revision). We include the relevant text here:

*"*The same group of participants who performed experiment 2 also performed experiment 3 (n = 36). We compared the two similar conditions (block 1 from experiment 3 with full coherence and block 1 from experiment 2 with equal extrinsic rewards), which differed in that choosing the *forced* option resulted in the obligatory selection of the same fractal (experiment 2) or one of two fractals randomly selected by the computer (experiment 3). We found that choice preference was similarly high (mean for experiment 3 = 69, bootstrapped 95% CI = [58, 78], and for experiment 2 = 68, 95% CI = [59, 76]) and did not differ significantly (mean difference (experiment 2 – experiment 3) = -0.013, bootstrapped 95% CI = [-0.106, 0.082], p = 0.792), indicating that subjects' choice preference in experiment 2 was not related to lack of action variability following *forced* selection per se. Moreover, we found that choice preference in these two blocks was significantly correlated (Spearman's r = 0.538, bootstrapped 95% CI = [0.186, 0.779], p = 0.003), highlighting a within-subject consistency in choice preference.*"*

16) I wonder how the optimistic learner can be fully distinguishable from the pessimistic one as the former is embedded in the later. Can the author keep the pessimistic model and compare it to a version with beta = 0 and to another version with beta = 1? That would allow the authors to show that whether the best model is more optimistic, more pessimistic, or in between.

The reviewer is right that the optimistic learner is contained within the pessimistic learner. Indeed, the β-pessimist is identical to a Q-learner when β = 1. To distinguish between them we fit the models with all the target policies to each subject, and already performed model selection using Schwarz's Bayesian Information Criterion (BIC). Since the β-pessimistic model includes an extra parameter, it is additionally penalized when it produces the same prediction as the optimistic model (i.e., when β = 1), and would not be selected. What was missing from the manuscript before is the actual value of β estimated when the pessimistic model is selected. These parameter values are now included in Supplementary Tables 1 and 2 and new Supplementary Figure 5, where one can see most β-pessimistic subjects weight the worst possible outcome as much if not much more than the best possible outcome. It was an oversight on our part not to include this in the original manuscript, and we now also mention this in the main text (p.39).

17) I suggest to add lines between each participant dot across all condition in the figure similar to figure 2B to provide the reader with an idea about preferences stability.

This is a good suggestion, and we tried several figure versions plotting lines for each subject. We could not find a clear illustration that allowed tracking individual subjects. Therefore, we now include additional figures plotting free choice preference across the different conditions for each subject (Sup. Fig. 1 for experiment 1 and Sup. Fig. 3 for experiments 2-3). We reproduce these below for convenience.

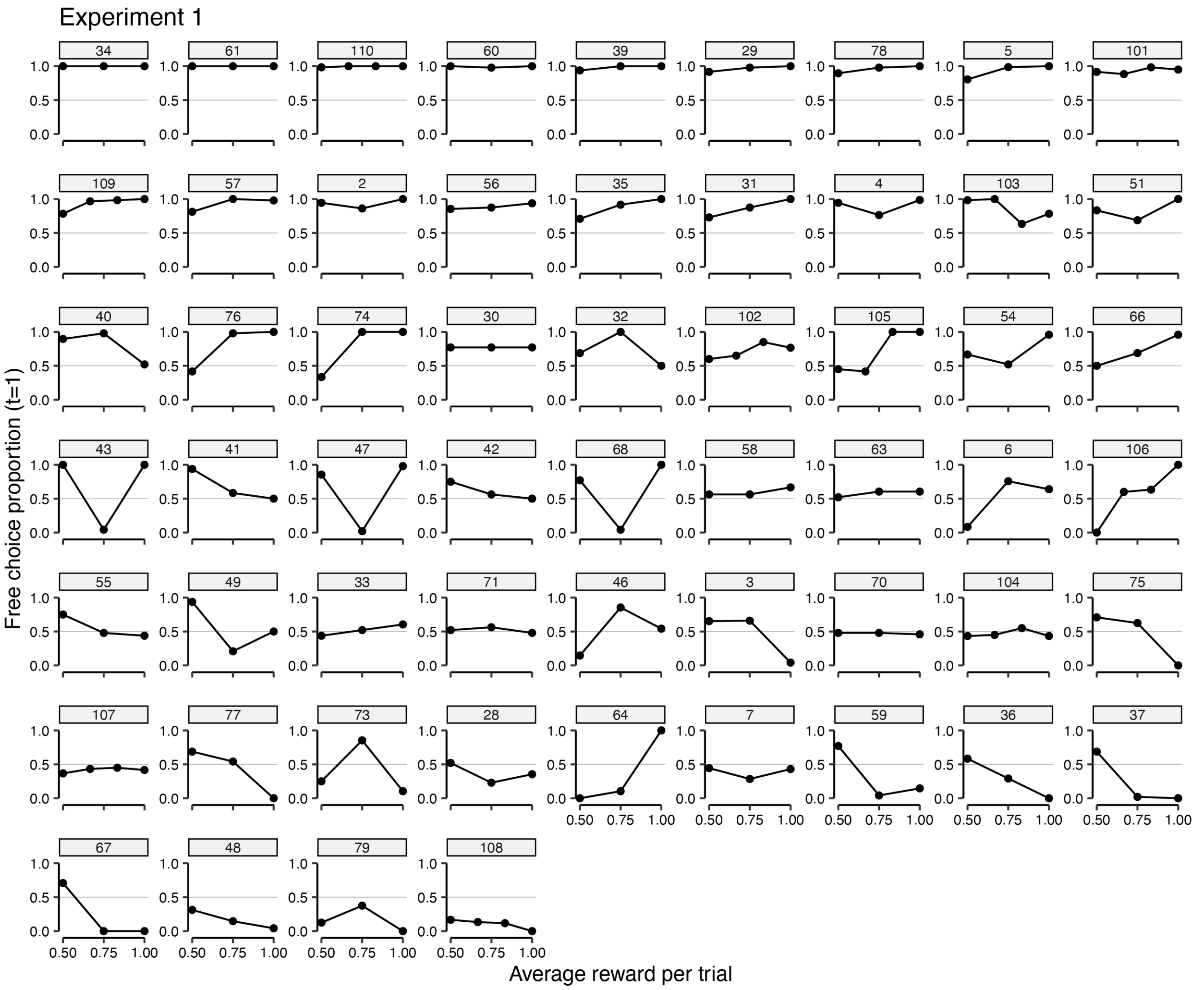

Supplementary Figure 1. First-stage free choice proportions for each subject in experiment 1. Subjects are ordered from left to right, top to bottom according to average free choice proportion (matches ordering of Supplementary Table 1).

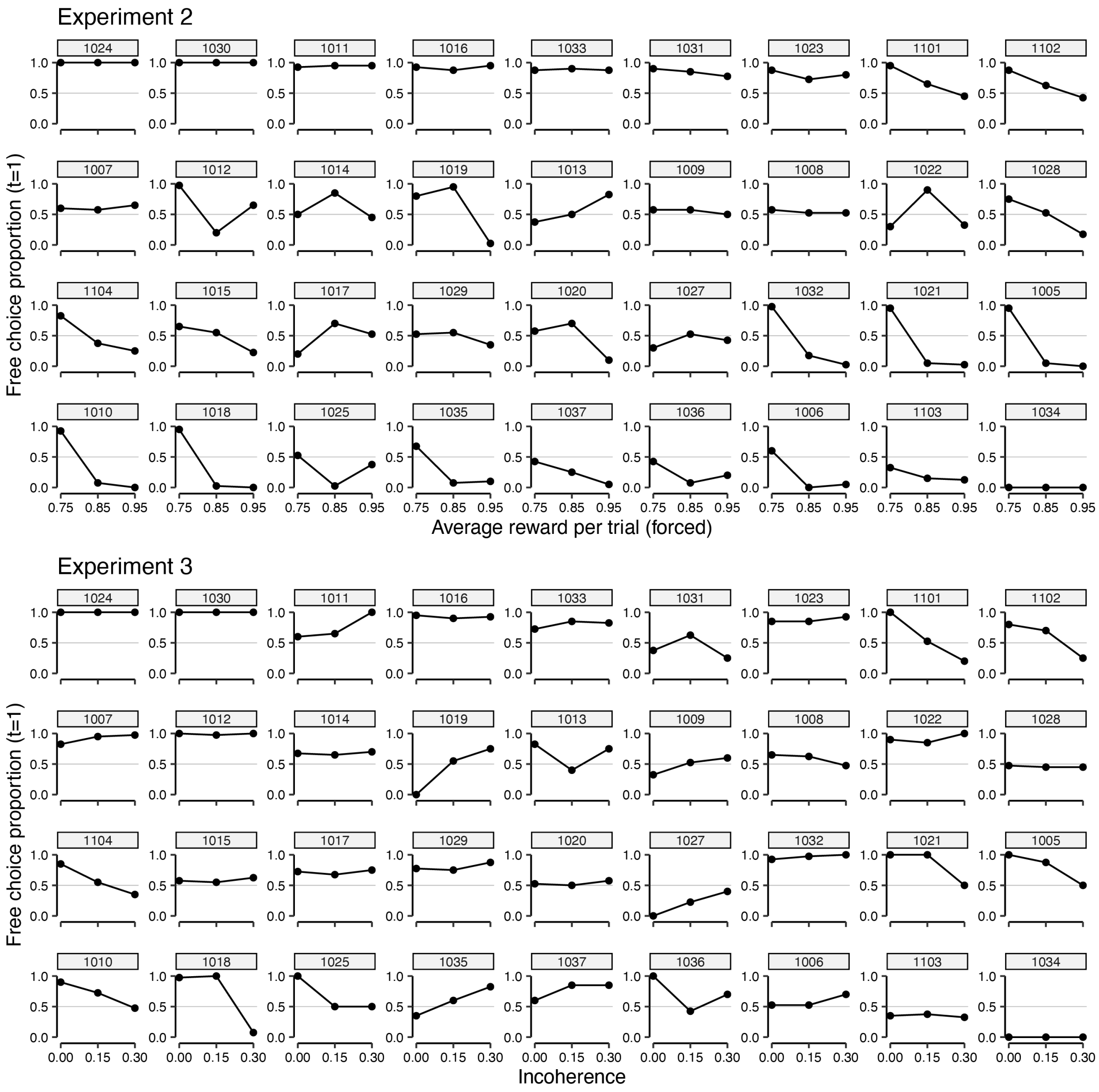

Supplementary Figure 3. First-stage free choice proportions for each subject in experiments 2 (upper panels) and 3 (lower panels). Subjects are ordered from left to right, top to bottom according to average free choice proportion in experiment 2 (matches ordering of Supplementary Table 2).

18) How were the sample sizes determined?

The sample size was chosen based on previous reward-guided decision-making studies using similar two-armed bandit tasks and probabilistic choices (Chambon et al, NHB 2020; Palminteri et al, Plos CB 2017; Palminteri et al, Nat. Comm. 2015). This information is available p.31 of the revised manuscript.

<u>Reviewer #3:</u>

In this paper, Munuera and colleagues report results from three experiments using a two-stage decision task that aims to computationally characterize peoples' preference for making choices. In Experiment 1, they found that even after learning the reward probabilities associated with second-stage choice options, participants demonstrated a consistent preference for choosing to make their own second-stage choice, rather than being forced to choose a maximally rewarding option. In Experiment 2, they found that participants continued to prefer to make their own second-stage choices, even when the forced choice option was more rewarding. And in Experiment 3, they demonstrated that in less controllable environments, when actions did not deterministically lead to expected states, participants' preference for making free choices was reduced.

Overall, I think the experiments nicely demonstrate people's preference for making choices, but many prior studies (which the authors cite) have demonstrated similar effects. The coherence manipulation in Experiment 3 is interesting and more novel, but I found the results difficult to interpret and the explanation the authors put forth for the effects overly speculative. Below, I lay out my concerns in more detail.

We thank the reviewer for her/his positive and constructive comments, and we have endeavored to address them all. We provide new evidence supporting our interpretation of the results from experiment 3 but we would like to emphasize that, while preference for free choice as been previously demonstrated, none of these studies had dissociated the intrinsic motivation for choice opportunities from learning about extrinsic rewards, tested any of the competing hypotheses explaining free choice preference, nor attempted to explain (or show) the intra- and inter-individual variability inherent to choice preference. Thus, in addition to the novelty of experiment 3, we strongly believe that experiments 1 and 2 also provide key and novel insights about the behavioral mechanisms underlying choice preference.

I think the authors' hypothesis that participants might be 'seeking personal control' in response to the incoherence of the environment is very interesting. However, I found it difficult to follow their reasoning for why the specific pattern of observed results supported this hypothesis. For example, it is not clear to me why participants' were necessarily "seeking personal control" by repeating their target selections regardless of reward outcomes on incoherent trials. It seems like an equally (or more) compelling explanation for these results is that participants rationally discounted the outcomes of events that were not caused by their own choices.

Thank you for bringing this point to our attention. We believe that the reviewer's interpretation is not exclusive of the one we give in the manuscript: discounting the outcomes of events that were not caused by one's own choices, even though a choice was made, is a way for participants to maintain the consistence of their beliefs in their instrumental capacities – that is, voluntary actions are generally followed by consequences that can be reliably predicted. What our data suggest is that this belief may be a default, and that the free-choice preference observed in our experiments instantiates this default belief.

As we mention in the Discussion, this default control belief has been described in the literature under many terms and concepts – whether self-efficacy (Bandura), sense of agency (Haggard), perceived behavioral control (Ajzen) or internal locus of control (Rotter). What has been widely documented is that when this control belief is threatened or challenged, subjects develop various strategies to restore it (Brehm and Brehm, 1981). Note that a variety of compensatory strategies for reduced control has been documented in humans: for example, individuals experiencing loss of control are more likely to use placebo buttons—to repeatedly press buttons that have no real effect—or to see images in noise or to perceive illusory correlations, etc. We suggest that discounting of choice outcomes is an occurrence of such a compensatory strategy: participants discount choice outcomes to compensate for the reduction in control induced by target swaps,

consistent with the broader psychology of control maintenance and threat-compensation literature (see Landau, Kay & Whitson, 2015, Psych Bull, for a review).

Repeating the incoherent free trials would thus simply be the corollary of discounting the outcomes of this trial. Note that this repetition is more than simple perseveration because it occurs specifically when the subject performs a voluntary action (free choice) and witnesses an incoherence between that voluntary action and its outcome. In this sense, it is reminiscent of the "try-and-try again" effect already observed in similar reinforcement learning studies measuring individuals' sense of agency in sequences of trials alternating free and forced choice (di Costa et al., 2018) or the "choice streaks" exhibited by participants when making decisions under uncertainty (Alméras et al., 2022). Thus, when the situation is uncertain, or the feedback from their action is questionable or contradicts what they believe, participants try again and repeat the same choice until they feel they have gathered enough evidence to make an informed decision and change or not change their mind (Rouault et al., 2022). Similarly, the repetition of choices in Experiment 3 suggests that participants are reluctant to update their belief about their personal control and repeat the same action to test it again.

Note that we do not claim that this compensatory strategy, which results in the repetition of incoherent trials, is consciously implemented by the participants. In this sense, we recognize that some of our formulations in the manuscript may have been misleading, or at best confusing. We have therefore deleted or reworded all occurrences suggesting a deliberate or conscious strategy:

Results section, p.19:
"The sustained repetition of free selections across the different levels of incoherence suggests that subjects may have been acting as if to maintain control over the task through self-determined 2nd-stage choices."

Results section, p.20:
"Finally, an alternative strategy could rely on maintaining control in situations where control is threatened by adverse events (e.g., action-outcome incoherence). Subjects would thus ignore any choice outcome from trials with reduced controllability (incoherent trials) and repeat their initial choice selection (Fig. 7B, fourth panel)."

"Results of the stay behavior during 2nd-stage choice following free selection suggests that subjects consider the source of reward when choosing between the different fractal targets (Fig. 7C)."

"We suggest that this strategy reflects an attempt to maintain a consistent level of control over the environment at the expense of the task goal of maximizing reward intake."

Discussion section, pp.28:
"Thus, subjects appeared to ignore information about action-state-reward contingencies that was externally derived, and instead appeared to double down by repeating their past choice as if to maintain some control over the task. This behavior is consistent with many observations suggesting that when individuals experience situations that threaten or reduce their personal control, they implement compensatory strategies to restore their control to its baseline level(54,55)."

We also made the following changes to the abstract, Introduction, Results, and Discussion sections:

Abstract:
"When deciding between options that do or do not lead to future choices, humans often choose to choose. We studied choice seeking by asking subjects to decide between a choice opportunity or performing a computer-selected action. Subjects preferred choice when these options were equally rewarded, even deterministically, and were willing to trade extrinsic rewards for the opportunity to choose. We explained individual variability in choice seeking using reinforcement learning models incorporating risk sensitivity and overvaluation of rewards obtained through choice. Degrading task controllability diminished choice preference following forced actions, although willingness to repeat selection of choice opportunities remained unchanged. In choices following these repeats, subjects were sensitive to rewards following freely chosen actions, but ignored outcomes associated with reduced controllability, in a manner consistent with a desire to maintain personal control over

outcomes. Choice seeking appears to reflect the intrinsic need for personal control, which competes with extrinsic reward properties and external information to motivate behavior."

Introduction, p.4:
"Finally, in the third experiment, we sought to test whether choice preference was motivated by personal control beliefs. We manipulated the controllability of the task and found that subjects' willingness to repeat a free choice was not affected by the lack of objective controllability over reward outcome. Importantly, subjects were sensitive to past rewards only in controllable trials, where state outcomes could be attributed to self-determined choice. In uncontrollable trials, however, subjects ignored reward outcomes and repeated their initial choice. We suggest that choice repetition in the face of uncontrollability reflects a strategy to compensate for reduced control over the environment, consistent with the broader psychology of control maintenance. Together, our results support the hypothesis that human preference for choice opportunities derives from the intrinsic motivation for personal control."

Discussion, p.28:
"Interestingly, when subjects selected the free option, the subsequent choice was sensitive to the past reward when the terminal state (the selected target) was coherent, and the reward could therefore be attributed to the subject's action. In contrast, subjects' choices were insensitive to the previous outcome when the terminal state was incoherent. Thus, subjects appeared to ignore information about action-state-reward contingencies that was externally derived, and instead appeared to double down by repeating their past choice as if they sought to maintain some control over the task. This behavior is consistent with many observations suggesting that when individuals experience situations that threaten or reduce their personal control, they implement compensatory strategies to restore their control to its baseline level(54,55). A variety of compensatory strategies for reduced control has been documented in humans. Thus, individuals experiencing loss of control are more likely to use placebo buttons–to repeatedly press buttons that have no real effect–to see images in noise or to perceive correlations when there are none (Whitson and Galinsky, 2008). We suggest that discounting of choice outcomes in incoherent trials, where personal control is threatened by unpredictable target swaps, is an occurrence of such a compensatory strategy. When faced with uncontrollability, participants would ignore any choice outcome and repeat their initial choice to compensate for the reduction in control, consistent with the broader psychology of control maintenance and threat-compensation literature (see Landau, Kay & Whitson, 2015, for a review).

Repetition of the initial choice in incoherent trials is more than mere perseveration (i.e., context-independent repetition) because it occurs specifically when the subject performs a voluntary (free) choice and witnesses an incoherence between that choice and its outcome. In this sense, it is reminiscent of the "try-and-try again" effect already observed in similar reinforcement-learning studies measuring individuals' sense of agency in sequences alternating free and forced choice trials (di Costa et al., 2018) or the "choice streaks" exhibited by participants when making decisions under uncertainty (Alméras et al., 2022). Thus, when the situation is uncertain, or the feedback from their action is questionable or contradicts what they believe, participants try again and repeat the same choice until they have gathered enough evidence to make an informed decision and change or not change their mind (Rouault et al., 2022). Similarly, the repetition of choices in Experiment 3 suggests that participants are reluctant to update their belief about their personal control and repeat the same action to test it again.

Finally, it should be noted that this strategy of ignoring choice outcomes from uncontrollable contexts is at odds with a pure model-based strategy(41), where an agent could exploit information about action-state-reward contingencies whether it derived from their own choices (controllable context) or from the environment or another agent (uncontrollable context) (…)"

One final point that we think is important: we argue that repetition is a response to certain features of experiment 3 that reduce control (the target swaps), which participants compensate for by repeating the initial selection. Such compensation is not necessarily irrational, as the participants were not given any specific information about the target swaps. In such a context of uncertainty, they therefore have no particular reason to update their beliefs about their control abilities but would do better to reject the incoherent trial and "try again". This observation raises the interesting question of how much disconfirming evidence is needed for subjects to update their control beliefs, with some

evidence suggesting that it takes more disconfirming evidence to change beliefs in controllable contexts than in non-controllable contexts (Weiss, Chambon et al., 2020, Nat Comms). One prediction that can be drawn for the present study is that the extent to which people repeat incoherent free trials is a function of the strength of their control belief: the more they believe they are in control of the task environment, the more they will need to repeat (i.e., accumulate disconfirming evidence) in order to update this belief. We believe this question can be addressed in future follow-ups directly testing associations between belief in personal control and the size of repetition sequences.

Added references:

Landau, M. J., Kay, A. C., & Whitson, J. A. (2015). Compensatory control and the appeal of a structured world. Psychological bulletin, 141(3), 694.
Whitson, J. A., & Galinsky, A. D. (2008). Lacking control increases illusory pattern perception. Science, 322(5898), 115-117.
Di Costa, S., Théro, H., Chambon, V., & Haggard, P. (2018). Try and try again: Post-error boost of an implicit measure of agency. Quarterly Journal of Experimental Psychology, 71(7), 1584-1595.
Alméras, C., Chambon, V., & Wyart, V. (2022). Competing cognitive pressures on human exploration in the absence of tradeoff with exploitation. PsyArXiv, doi:10.31234/osf.io/9qpuz.
Rouault, M., Weiss, A., Lee, J. K., Drugowitsch, J., Chambon, V., & Wyart, V. (2022). Controllability boosts neural and cognitive signatures of changes-of-mind in uncertain environments. ELife, 11, e75038.

In addition, it is unclear how the computational account put forth for the findings from Experiment 1 and 2 can be extended to account for the findings from Experiment 3. The authors found that the majority of subjects in Experiment 1 and 2 were best fit by an optimistic reinforcement learning model with a free choice bonus that simply inflated the value of rewards obtained after choice. However, simply inflating the value of the rewards obtained from chosen options does not seem like it can explain the findings from Experiment 3 — instead, the model would need to estimate the coherence of the environment, and/or the probability that rewards are the result of one's own actions, and modulate the bonus accordingly. Thus, while I found Experiment 3 interesting, I was unsure how to interpret the findings within the framework set up by the rest of the paper.

This is a very interesting point raised by the reviewer that we did not model here. We agree with this reviewer that the two novel elements introduced by our modelling of experiments 1 and 2—the free choice bonus and the risk aversion—do not obviously predict the pattern of 2nd-stage choices we observed in experiment 3. However, we note that by design all terminal actions in experiment 3 lead to the same expected reward, and a free choice bonus would still be necessary (in addition to some internal model of self-determined actions) to generate choice preference. That is, subjects who veridically measure extrinsic outcome rates would be indifferent to free and forced options even if they estimated which rewards resulted from their own actions. Discriminating between different models that could capture aspects of environmental structure is outside the scope of our study. We believe that to sharply arbitrate between these models requires specific task designs, which is something we plan to do in a full follow-up study. Consistent with this reviewer's remark, our hypothesis is that the effect of controllability on how subjects treat choice outcomes could be captured by a component in addition to the free choice bonus, namely a model-based controller that estimates transition probabilities (e.g., Daw and colleagues), models capturing hybrids between model-based and model-free control (including successor representations), and/or by an inferential (Bayesian-like) component that rationally assigns credit to different possible sources of feedback based on controllability cues. Thus, while the details of the environmental model remain to be clarified, what our data strongly suggests is that outcomes of self-determined actions are distorted, and this must be accounted for in future experiments. We now discuss this briefly in the Discussion section (pg.29):

"Finally, it should be noted that this strategy of ignoring choice outcomes from uncontrollable contexts is at odds with a pure model-based strategy(41), where an agent could exploit information

about action-state-reward contingencies whether it derived from their own choices (controllable context) or from the environment or another agent (uncontrollable context). Rather, it is consistent with work showing that choice-seeking could emerge when self-determined actions amplify subsequent positive reward predictions(5,36), and more generally with the notion that events are processed differently depending on individuals' beliefs about their own control abilities. Thus, positive events are amplified only when they are believed to be within one's personal control, as opposed to when they are not(36), or when they come from an uncontrollable environment(58).*"*

I also wondered about alternative computational accounts for the findings from Experiments 1 and 2. Here, the authors implemented the enhanced valuation of free choice outcomes via a bonus added to the reward outcome. It seems like other computational mechanisms could also explain these effects. In particular, prior studies have found that different higher learning rates for positive vs. negative outcomes following free choice can explain the preference. Is this model distinguishable from the choice bonus model described here?

Thanks for pointing this out. Prior studies have shown that different learning rates for positive and negative prediction errors can account for overvaluation of cues learned in free choice versus forced selection contexts (e.g., Chambon et al., 2020). Our decision to use a scalar bonus was motivated by the results from Chambon and colleagues (their Discussion as well as Supplementary Methods and Results), who showed that a model that directly altered the free choice outcomes was able to replicate the results of different positive and negative learning rates. This indicates that these mechanisms mimic each other and are difficult to distinguish in practice, and that specifically designed tasks are probably necessary for definitive claims about when one or the other of these mechanisms can be ruled out. Also, in order to produce choice preference, differential learning rates would have to be fit for both the free and forced options, which would greatly increase the number of parameters in our task (to handle 1st and 2nd stages). Finally, the free choice bonus is conceptually easier to apply for understanding prior studies where choice preference is demonstrated where there is no obvious learning (e.g., one-shot questionnaires about fictitious outcomes). We have added text to highlight these points p.25-26.

In addition, it seems as though an alternative account could be that making the choice is in and of itself rewarding. Rather than inflating the value of the outcome obtained through free choice, an alternative model could add a bonus directly to the first-stage free choice option. In other words, do participants learn more from free vs. forced choices or simply prefer them?

We thank the reviewer for posing this question. We understand "bonus directly to the first-stage free choice option" to mean an offset or bias added to the free state-action Q-value when a 1st-stage decision is made. This will generate free choice preference but will not enter directly into subsequent value updates (hence it will not directly affect Q-value learning). So concretely, let a^1 *represent the action that selects the free option, then a softmax policy biased towards free choice is:*

$$\pi(s, a^1) = \frac{\exp\left([Q(s, a^1) + \mathrm{bias}]/\tau\right)}{\exp\left([Q(s, a^1) + \mathrm{bias}]/\tau\right) + \exp\left(Q(s, a^2)/\tau\right)}$$

The key difference we see between the outcome bonus as we have implemented versus a biased softmax policy is that the latter does not vary over time. Implementing a biased softmax policy is potentially useful when the bias on decision making is stable, as is the case for perceptual distractors (e.g., a flanker, see Sidarus, Chambon & Palminteri, 2019). Our initial decision to implement a free choice outcome bonus was directly motivated by prior experiments showing that cues associated with reward contingencies learned in a free choice context were overvalued compared to cues associated with the same reward contingencies learned in a forced choice context (Cockburn et al, Neuron 2014; Chambon et al, NHB 2020).

However, we appreciate that it is still interesting to know whether one model fits the subject data better. Therefore, we fit 24 additional models (one for each outcome bonus variant, replacing the outcome bonus with a policy bias) to each subject's data from experiments 1 & 2. First, we directly compared model evidence across all model pairs that were identical except that one was fit with a bonus and the other with a bias (2256 model pairs). This yields a distribution of differences (ΔBIC) with a mean = -7.76. This can be interpreted as an approximate Bayes factor that is strongly in favor of the outcome bonus model (2,3). We next examined the winning model for each subject. For 72% (68/94) of subjects the winning model was the same model that was selected before including the models with bias. Finally, we focused specifically on subjects where the winning model included a bonus or a bias (n = 63). We found that the best models for 59% (37/63) of subjects maintained a free choice outcome bonus when this parameter was originally included in the winning model, while for 30% (19/63) of subjects the best model switched from including an outcome bonus to including a bias instead. 11% (7/63) of subjects who did not have an outcome bonus originally ended up retaining a bias in the expanded model space. This indicates that the outcome bonus model fits best for most subjects. However, the preceding breakdown was based on ΔBIC of any magnitude and does not capture the strength of evidence. Therefore, we plot the ΔBIC (bonus – bias, lower is better) between the winning model and the next best model with a bonus or a bias (Supplementary Figure 7). This ΔBIC distribution has a mean = -13.8, which is strongly in favor of our original outcome bonus model. Moreover, there were 24/63 subjects where $|\Delta BIC|>20$ *(mean = 41.2), which constitutes very strong evidence in favor of the outcome bonus model, versus 5/63 with the same strength of evidence in favor of the biased softmax policy (mean = 17.0). Taken together, these results indicate that a free choice outcome bonus fits the data better than a softmax bias, suggesting that most subjects update free and forced option values rather than applying a static bias. We have included the figure below and elements of the preceding response in the supplementary materials (Supplementary Figure 7 and associated text, pp.3-4 of Supporting information), and text in the results (p. 14).*

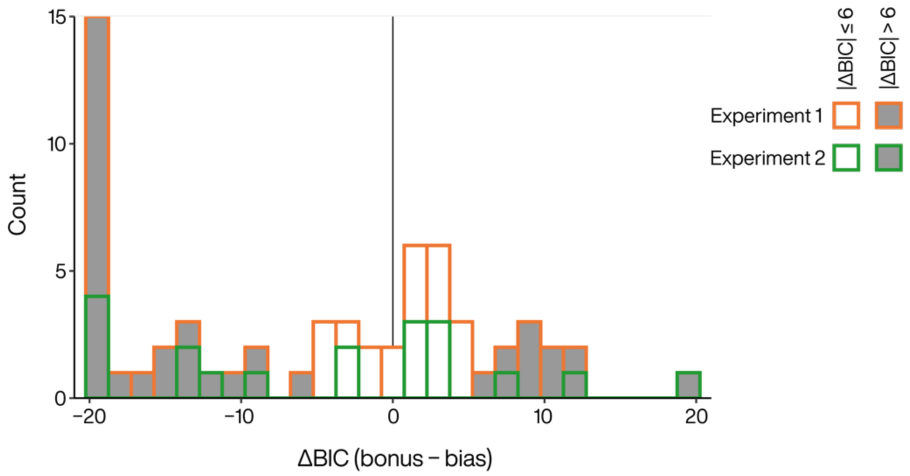

Supplementary Figure 7. Strength of evidence supporting free choice bonus model versus biased softmax policy model. Values along the abscissa represent the difference in BIC scores between the winning model and the next best model with a bonus or a bias for all subjects where the winning model included one of these parameters (*n* = 63). Differences less than -20 are included in the leftmost bin (*n* = 14) and differences greater than 20 are included in the rightmost bin (*n* = 1). Shaded bars indicate data where $|\Delta BIC| > 6$, corresponding to strong evidence in favor of the bonus model (negative values) or the bias model (positive values).

Though perhaps outside the scope of the present work, one way to address this question might be to have an additional phase of the task where participants must choose between the forced choice fractal and the high-value free choice fractal. Does the high-value free choice fractal remain more valuable to participants?

As noted above we implemented a free choice bonus based by prior experiments showing that cues associated with reward contingencies learned in a free choice context were overvalued compared to cues associated with the same reward contingencies learned in a forced choice context (e.g., Cockburn et al, Neuron 2014; Chambon et al, NHB 2020). Our modelling is consistent with these prior publications. We did not evaluate this overvaluation by asking subjects to directly compare the forced and free fractals. However, we did ask subjects to estimate reward probabilities of the 2nd-stage fractals in both the free and forced options. As we detail immediately below, we found that subjects accurately estimated both free and forced fractal reward rates, and that choice preference was not due differential distortion of these probabilities.

Similarly, do participants have accurate explicit knowledge of the reward probabilities associated with the fractals, or are their estimates for those following free choice inflated?

This is a great question that was also posed by Reviewer 2. We actually collected data relevant to probability estimation and thank the reviewers for the opportunity to present it. We specifically asked to the subjects in the experiment 2 to estimate the probability of extrinsic reward for each 2nd-stage fractal image. They did so by placing a cursor on a visual scale from 0 to 100 after completing each block of trials. We found that the subjects were relatively accurate at estimating the reward probabilities for fractals in both free and forced trials, with mild underestimation (overestimation) at higher (lower) reward probabilities (estimated trend forced = 0.749, 95% CI = [0.683, 0.816], t = 22.2, p < 0.001; estimated trend free = 0.735, 95% CI = [0.640, 0.831], t = 15.2, p < 0.0001), which did not differ significantly between these trial types (estimated trend difference = 0.014, 95% CI = [-0.102, 0.130], t = 0.237, p =0.812). This suggests that preference for the free option was not due to differential distortion in estimating the frequency *of rewards. We think is quite novel information that we had not thought to examine before and we thank the reviewer for making this idea clear to us. We have integrated the preceding figure into the main text (Figure 4 in revision), with associated text (pp.11-12).*

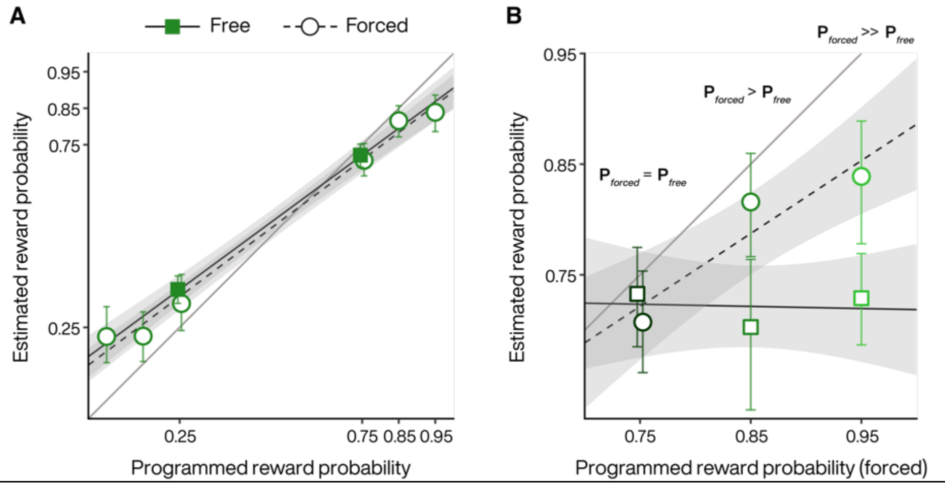

Figure 4. Subject estimates of extrinsic reward probabilities in experiment 2. **A**. Each circle (forced) or square (free) represents the average estimate of each 2nd-stage images experienced or viewed by the subjects across the three blocks. The estimates for fractals in the free option were averaged across the blocks but were similar both for the poorly rewarded targets (0.39, 0.35 and 0.32 for P_{forced} = 0.75, 0.85 and 0.95 respectively) and the highly rewarded targets (0.73, 0.70 and 0.73 for P_{forced} = 0.75, 0.85 and 0.95 respectively)). Black lines with shading indicate the estimated trends from a GLMM, with 95% CI. **B**. Same as in A but only for the highly rewarded targets (i.e., the most selected, see Fig. 3). Note that in contrast to A., the abscissa in B. refers to the maximal reward probability for the

forced option. P_{free} did not vary significantly (estimated trend = -0.019, 95% CI = [-0.331, 0.293], p = 0.902), consistent with the maximal reward probability for the free option being constant at 0.75. Black lines with shading indicate the estimated trends from a GLMM, with 95% CI.

A few minor concerns: It does not seem reasonable to initialize the Q value estimates at 0, since the actual outcomes values range from 0 - 1. Initializing at .5 seems less biased.

We thank the reviewer for this comment, which was also raised by Reviewer 2. Q-value initialization is indeed an important hyperparameter. In the machine learning literature, Q-values are frequently initialized at 0 when other mechanisms are incorporated to promote exploration (ε-greedy or softmax decision rules (1)). Without other exploration mechanisms, initializing Q-values at zero would indeed bias agents towards choosing whichever action led to the first reward, which could be non-optimal. Since we used the softmax decision rule we originally initialized the Q-values at 0 because, like initializing at 0.5, it indicates initial indifference between options (this decision rule considers only Q-value differences). However, Reviewers 2 and 3 raise the possibility that initializing at 0.5 might better represent the subjects' initial conditions and therefore provide a better fit to their data. To address this, we fit the whole set of models using initial Q-values = 0.5 (4512 model fits). Briefly, the outcome of our analyses is that initial Q-values = 0 is overall superior to initial Q-values = 0.5, and that using the model with initial Q-values = 0.5 for those individual subjects where it provides a better fit does not change our conclusions.

First, we directly compared model evidence across all models with initial Q-values = 0 and 0.5, which yields a distribution of differences (ΔBIC) with a mean (median) = -17.6 (-7.28). This can be interpreted as an approximate Bayes factor in favor of initial Q-values = 0 (2,3). We next examined this more closely by comparing the best model selected for each subject under each scenario (Figure SX A). Again, the evidence is in favor of initial Q-values = 0, with a mean (median) difference = -4.38 (-3.68) (Figure SX A, inset), which can also be observed using Tjur's (4) R^2 to measure model fit (Figure SX B). For 63% of subjects (59/94), the model with initial Q-values = 0 is selected (ΔBIC<0). In 40% of the cases (14/35) where the model with initial Q-values = 0.5 is selected (ΔBIC>0), the selected model was structurally identical to that selected for initial Q-values = 0 (i.e., same target policy, presence/absence of free choice bonus, etc.). In the remaining 21/35 subjects, 8 exhibited ΔBIC>6, which constitutes strong evidence (2) in favor of initial Q-values = 0.5. For these, we examined our main parameter of interest, the free choice bonus, and we found that this parameter was retained in every case where it was retained in our initial fits with initial Q-values = 0 (n=6). Finally, we compared the free choice bonus for all models where it was retained for both initial Q-values = 0 and 0.5 (n = 51) and found that the estimated bonus parameters were significantly correlated (r = 0.838, p < 1e-6).

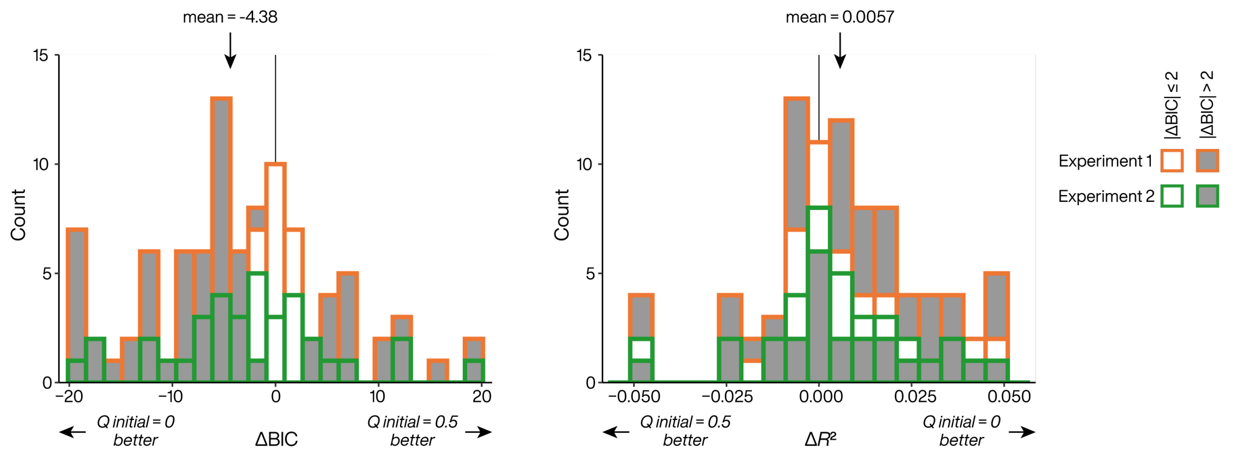

Supplementary Figure 8. Comparing models fit with initial Q-values equal to 0 or 0.5. The left panel shows **Δ**BIC scores (negative values favor initial Q-values = 0), and right panel shows delta Tjur's $\boldsymbol{R}^2$(positive values favor initial Q-values = 0).

Taken together, these analyses indicate that, under the conditions of our task, the evidence supports our original choice of initial Q-values = 0. However, some subjects were indeed better fit with initial Q-values = 0.5. We have therefore revised the manuscript to select the best Q-value initialization for each subject. This is detailed in the methods (p. 39 in the main new manuscript and pp.4-5 of the new Supporting information). Also, new Figure 5 (originally Fig. 4), supplementary Figure 6 (originally Sup. Fig. 2) and new Supplementary Figure 8 have been updated to reflect these minor changes, which do not alter any of our conclusions.

While the description of the task in the methods section at the end of the article is very clear, certain information that was critical for interpreting the results was not presented upfront. In particular, it would be helpful to know how long the training phase was, whether participants were instructed about the relationship between the reward probabilities, and how many participants completed Experiment 1.

Indeed, this information was in the methods in the original manuscript, and we agree that it is worth repeating in the results. We have added it in the results section before the Fig.1 (Task design, p5).
