# Supplementary material for: Intrinsic motivation for choice varies with individual risk attitudes and the controllability of the environment
Source: PLoS Comput Biol. 2023 Aug 11;19(8):e1010551. doi: 10.1371/journal.pcbi.1010551 (PMC10479909; doi:10.1371/journal.pcbi.1010551)
Supplement: Attachment — Submitted filename: Munuera_etal_response_to_reviewers.pdf [file pcbi.1010551.s003.pdf]

We thank the reviewers and editors for their feedback and comments during the review process.

We acknowledge that the personal control hypothesis is not straightforward, and so in the revised version of the manuscript we have made consistent changes throughout to emphasize both its limitations and speculative nature. We also now give more balanced emphasis to our other novel findings, including the dissociation observed between optimistic and pessimistic attitudes, both in abstract and discussion of the paper and, as suggested by the editor, in the title which we have changed to focus on findings.

Because personal control is a well-established concept in the literature and we remain convinced that it is supported by our results, we have chosen to retain it as an element of discussion. We have carefully edited the discussion to clearly distinguish what we found from how we interpret the findings and have added further clarification to the points raised by reviewer 2 in this round of reviews (detailed below).

Below, in italics, are our specific responses to the reviewers.

Reviewer #1: The authors have gone above and beyond to address my previous comments and I have no further suggestions. In terms of adding Figure R1 (RT) into the supplement, I think it would be useful, as it helps bolster the claims of the paper. However, I will leave the decision to include it up to the authors and the editor given space constraints and other considerations.

We thank the reviewer for her/his very valuable comments during the review process. We have included this result on p. 22 of the revised manuscript and the associated figure is now available as a new supplementary item (Figure 8).

Reviewer #2: While I acknowledge that the authors put efforts in addressing my (and very similar reviewer 3's) concerns, I still do not see any data supporting the title and the conclusions.

What is straightforward in the paper is not new (experiment 1 & 2), and what is new is not straightforward (experiment 3). In particular, the direct test of the hypothesis that "Choice seeking is motivated by the intrinsic need for personal control" is that free choice preference should be altered by lower level of controllability. That would show that when people are in control in free choice, they choose free choice, but when they are no longer, they equally prefer forced choice. This test was not reported in the first version of the manuscript and is in fact not significant in the revised version.

We acknowledge the reviewer's point of view, but we respectfully disagree regarding the novelty of our paper. As we mentioned in the previous round of review, although free choice preference has been demonstrated before, none of these studies had dissociated the intrinsic motivation for choice opportunities from learning about extrinsic rewards, tested any of the competing hypotheses that could explain free choice preference, or attempted to show or interpret the intra- and inter-individual variability inherent to choice preference. We address all these open questions using Experiments 1 and 2 and develop a computational model of choice seeking that represents an important step towards understanding the intra- and inter-individual variability of choice preference. Thus, the results associated with Experiments 1 and 2 provide key and novel insights about the behavioral mechanisms underlying choice preference and intrinsic motivation in general.

Regarding Experiment 3, we have included the information requested in the last round of review. We also re-emphasize that while the average choice preference did not decline significantly with increasing incoherence, subjects did in fact significantly modify their behavior

when confronted with incoherence (by decreasing their likelihood of switching to a free choice following selection of the forced option). We have added text in the abstract, introduction, and discussion, and are confident that this is clear throughout the revised manuscript. Our personal control interpretation follows from the further analysis of 2nd-stage choices, which we suggest explains choice at both stages of our task. We appreciate that the original title and abstract focused on our personal control interpretation, and so in the revised version of the manuscript we have made consistent changes throughout so that any discussion surrounding the results of Experiment 3 is appropriately qualified by interpretational limitations and placed in clear context of concrete alternative interpretations (more below) as well as relative to our factual results. We have also changed the title and abstract to emphasize more representatively our other novel findings.

While the fact that incoherent choices are repeated regardless the outcome can be interpreted as a compensatory mechanism to feel in control when incoherency is high, it can be equally interpreted differently. For example, people may be model based learner, but they may attribute positive outcomes to their credit and they would repeat the same action. (Moreover, I think that the parametric modulation of this effect remains to be tested: the higher the incoherence level, the stronger the compensation should be.) It is anyway not a direct proof that "Choice seeking is motivated by the intrinsic need for personal control".

Importantly, our results cannot be interpreted in a purely model-based manner, as we have already discussed in the paper (p.30). To account for our data, we proposed a compensatory strategy where subjects detect incoherence, and proceed by repeating their previous self-determined choice. To make the argument even clearer, we now clarify that ignoring choice outcomes after incoherent trials applies to both positive and negative outcomes, which a model-based strategy cannot easily account for. Moreover, our interpretation does not claim that repetition should be parametrically modulated by incoherence level, as it simply depends on detecting when an action does not match the expected stimulus outcome.

The reviewer suggests a modified model-based strategy that additionally includes the attribution of any positive rewards to an agent's credit. This explanation is less parsimonious than our proposal, as it requires two contradictory strategies to explain the same behavior: illusory self-attribution in the case of rewarded incoherent actions (the choice is repeated because subjects attribute positive outcomes to themselves, failing to detect incoherence and that the choice is wrongly reinforced) AND detection of incoherence when there is no reward (here, the choice is repeated because the subject understands that the choice is wrongly reinforced). This is contradictory, as either incoherence is detected (and illusory attribution is then impossible), or incoherence is NOT detected (and there is then no reason to repeat the choice after obtaining an unrewarded outcome). Nevertheless, to contrast our proposed strategy relative to this and other possibilities, we have included the reviewer's suggestion in the discussion:

P.30 of the new manuscript version:

"*Finally, it should be noted that this strategy of ignoring choice outcomes—**positive or negative**—from uncontrollable contexts is at odds with a pure model-based strategy(42), where an agent could exploit information about action-state-reward contingencies whether it derived from their own choices (controllable context) or from the environment or another agent (uncontrollable context). Rather, it is consistent with work showing that choice seeking could emerge when self-determined actions amplify subsequent positive reward prediction errors(5,37), and more generally with the notion that events are processed differently depending on individuals' beliefs about their own control abilities. Thus, positive events are amplified only when they are believed to be within one's personal control, as opposed to when they are not(37), or when they come from an uncontrollable environment(58). **A simple strategy***

consistent with the behavior we observed is one where subjects track rewards from self-determined actions but ignore rewards following target swaps that are incoherent with their actions. Alternatively, subjects may apply a modified model-based strategy where they attribute all positive outcomes to their credit, although here the high repetition rate would be due to illusory self-attribution on rewarded incoherent actions and model-based non-attribution on unrewarded incoherent actions. Key to both strategies, however, is that subjects modify their behavior based on recognizing when their self-determined actions produce incoherent outcomes."

Overall, I can only see three options here: the title not being supported by the data, the paper cannot be published as such (hence rejected); at least a pre-registered experiment 3' including more incoherent levels (e.g. up to 0.5) shows that free choice preference is altered when personal control is lower (so that the need for personal control can no longer be satisfied); the paper is reframed to focus on the interesting (but not directly related to personal control) experiment 3 result, which is no longer about free choice and personal control.

As noted above, we recognize that the maintenance of personal control hypothesis is not straightforward and therefore downplay it throughout the article, title and abstract included. We believe that all the changes together result in a balanced presentation of our novel findings, and for which we make appropriate and qualified interpretations.

In addition, I have other suggestions:

It remains unclear to me how the learning model with a bonus differs from a model with a choice bias. Can the authors report a qualitative signature of this model that a model with a bias could not capture? Model comparison is often relevant, but may be biased.

Details regarding choice bias versus free-choice bonus are available on p. 15-16 of our manuscript with further details included in the supporting information (p.3 and 4 for the explanation of differences, and Sup. Fig. 7 for the direct comparison between both). One key difference between the outcome bonus we implemented versus a biased policy is that the former enters into subsequent value updates to modify Q-values. Importantly, rewards were probabilistically delivered (except for the one deterministic condition in Experiment 1), meaning that a reward-prediction error learning rule could track local stochastic fluctuations in extrinsic rewards that could yield changes in free choice preference. A bias, on the other hand, can generate free choice preference but will not enter directly into subsequent value updates (hence it will not directly affect Q-value learning). Thus, a bias, which must occur at the 1st-stage to generate choice preference, cannot explain the results of experiments where actions taken in a free-choice context have higher value than actions taken in a forced-action context for the equivalent extrinsic reward (e.g., Cockburn et al., Neuron, 2014; Chambon et al., Nat Human Behav, 2021). On the other hand, a bonus as we have implemented can produce these prior experimental findings in addition to our own subjects' choice behavior. This is noted in the paper (p. 16):

"Our initial decision to implement a free choice outcome bonus was motivated by prior experiments showing that cues associated with reward contingencies learned in a free choice context were overvalued compared to cues associated with the same reward contingencies learned in a forced choice context (5,37)."

On that note, model recovery results could be performed (but even then, qualitative model validations are crucial).

We performed additional model recovery experiments for bonus and bias models (similar to how we performed model recovery in Supplementary Figure 4). We found that model confusion, p(fit|sim), was 0.89 for bias when the simulation was bias and 0.75 for bonus when the simulation was bonus. We found that model inversion, p(sim|fit), was 0.78 for bias when the fit was bias and 0.87 for bonus when the fit was bonus). These values are high and in a similar range as those we observed for model recovery of the other parameters of the models we used. Therefore, we are confident that the model comparison between bias and bonus models is reliable. We have included this information on p. 3 of the supplemental information in the section dedicated to comparing bias and bonus model fits.

In the same vein, I cannot explain why a q value initialised at 0 is better. If it is added as a free parameter, is it indeed estimated to be 0?

Details regarding Q-intialization are available on p. 39-40 of our manuscript with further details included in the supporting information (p. 4 and Sup. Fig. 9 for the direct comparison between initial Q=0.5 and Q=0). As we noted previously, Q-values are frequently initialized at 0 when other mechanisms are incorporated to promote exploration (ε-greedy or softmax decision rules). Sometimes an "optimistic" initialization is used, whereby Q-values are initiated positively. This promotes early exploration since any reward obtained will drive the corresponding Q-value closer to reality, while all other Q-values remain higher (thus increasing the likelihood the actions corresponding to these Q-values are selected in the future if the obtained rewards are sufficiently smaller than the initial Q-values). Eventually, the Q-values will converge to the correct action-values as the "optimistic" initial values are overwritten. This can be contrasted with a "pessimistic" initialization, whereby Q-values are initiated to a low value. The end effect here is that agents will tend to stick with initial actions if the obtained rewards are sufficiently larger than the initial Q-values. Our subjects who were better fit with initial Q=0 are therefore those subjects who were less exploratory and tended to stick with their initially rewarded actions. However, this was not absolute since action selection was determined using a softmax function to capture subject exploration (allowing sampling of other states despite pessimistic initialization), and rewards were probabilistic such that during learning subjects could easily obtain no reward (a situation where initial Q=0 would be correct). These two factors likely contribute to why the difference in R^2 values for initial Q=0.5 and Q=0 is quite small (<0.05 in absolute value; plotted in Supplementary Fig. 9), which indicates that Q-initialization within this range does not meaningfully impact model fits to behavior.

<u>Reviewer #3:</u> **The authors have done an excellent job responding thoroughly to my concerns as well as those raised by other reviewers. I believe the changes they made to the manuscript greatly improve its clarity, and the additional modeling analyses provide stronger support for their original conclusions. I believe this paper will make a valuable contribution to the literature and am happy to recommend its acceptance.**

We thank the reviewer for her/his very helpful comments during the review process.
